# Investigating the quality of extraction and quantification of bioactive compounds in berries through liquid chromatography and multivariate curve resolution

**DOI:** 10.1007/s00216-024-05474-8

**Published:** 2024-08-15

**Authors:** Thamani Freedom Gondo, Fang Huang, Nittaya Marungruang, Lovisa Heyman-Lindén, Charlotta Turner

**Affiliations:** 1 Department of Chemistry, Centre for Analysis and Synthesis, Lund University, P.O. Box 124, 22100 Lund, Sweden; 2 Department of Chemistry, Division of Biotechnology, Lund University, Lund, Sweden; 3 Aventure AB, Lund, Sweden; 4 Berry Lab AB, Lund, Sweden; 5 Department of Experimental Medical Science, Lund University, Lund, Sweden

**Keywords:** Berries, Degradation kinetics, Evolving factor analysis, Extraction selectivity, Multivariate curve resolution, Polyphenols

## Abstract

Berries are a rich source of natural antioxidant compounds, which are essential to profile, as they add to their nutritional value. However, the complexity of the matrix and the structural diversity of these compounds pose challenges in extraction and chromatographic separation. By relying on multivariate curve resolution alternating least squares (MCR-ALS) ability to extract components from complex spectral mixtures, our study evaluates the contributions of various extraction techniques to interference, extractability, and quantifying different groups of overlapping compounds using liquid chromatography diode array detection (LC-DAD) data. Additionally, the combination of these methods extends its applicability to evaluate polyphenol degradation in stored berry smoothies, where evolving factor analysis (EFA) is also used to elucidate degradation products. Results indicate that among the extraction techniques, ultrasonication-assisted extraction employing 1% formic acid in methanol demonstrated superior extractability and selectivity for the different phenolic compound groups, compared with both pressurized liquid extraction and centrifugation of the fresh berry smoothie. Employing MCR-ALS on the LC-DAD data enabled reliable estimation of total amounts of compound classes with high spectral overlaps. Degradation studies revealed significant temperature-dependent effects on anthocyanins, with at least 50% degradation after 7 months of storage at room temperature, while refrigeration and freezing maintained fair stability for at least 12 months. The EFA model estimated phenolic derivatives as the main possible degradation products. These findings enhance the reliability of quantifying polyphenolic compounds and understanding their stability during the storage of berry products.

**Graphical abstract:**

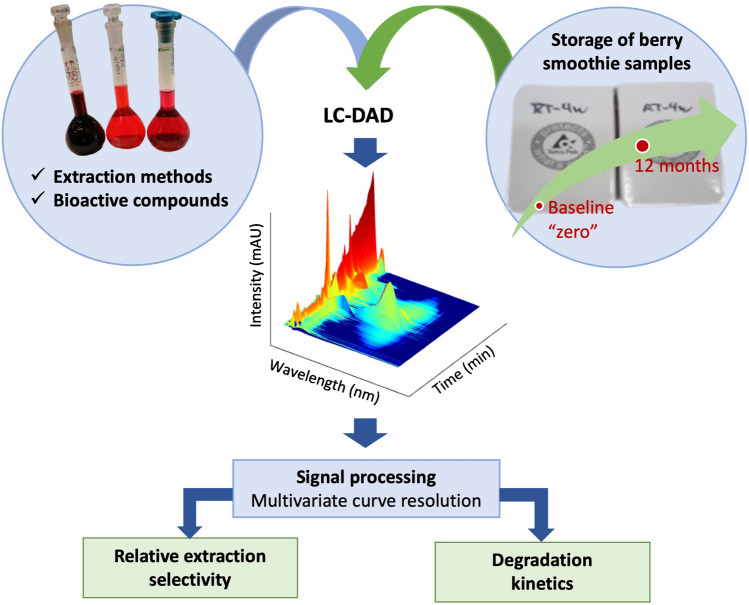

**Supplementary Information:**

The online version contains supplementary material available at 10.1007/s00216-024-05474-8.

## Introduction

Fruits serve as an important source of nutrients worldwide [1]. Berries in particular are a rich source of various compounds crucial for both food processing and nutrition. Despite the abundance of wild berries in the Swedish forests, the volume produced per annum remains underutilized. Phenolic compounds found in berries, such as phenolic acids, flavonoids, and anthocyanins, contribute significantly to their sensory properties, such as colors and taste [2, 3]. Anthocyanins in particular are the predominant phenolic composition of berries [4], comprising cyanidin, delphinidin, peonidin, pelargonidin, and petunidin, often in their glycosylated form. Beyond their sensory attributes, these compounds are recognized for their antioxidant properties. Such bioactive compounds have the ability to modulate the metabolic processes in biological system, suggesting their potential application in addressing human health. Some in vivo studies suggest that berries and their phenolic compounds may influence the intestinal microbiota, reducing low-grade systemic inflammation [5], improving cognitive function [6], and potentially mitigating metabolic dysfunction including obesity [7, 8]. Some studies have also reported microencapsulation of extracts of bioactive compounds for potential application as pharmaceutical ingredients [9]. Moreover, phenolic compounds are integral in preventing oxidation during food processing, highlighting their importance in maintaining product quality [10]. In the food industry, knowing the amounts of these compounds, as well as understanding the stability and degradation kinetics during storage, is one of the crucial factors pertaining to product quality. Additionally, as berries are gaining attention for positive health effects, it is crucial to monitor these bioactive compounds in berry products particularly intended for clinical trials. Therefore, storage conditions and time should be addressed, which often depends on the manufacturing process and conditions, hence may vary greatly between different products.

The complexity of berry-based products necessitates the development of robust analytical methods, especially for quantitative analysis. Chromatographic separation is commonly employed, aiming for high peak capacity and resolution. Nevertheless, challenges persist due to matrix complexity and the presence of interfering components, which can vary between samples, thereby compromising the method’s robustness. Sample preparation, as the initial step before chromatographic separation, significantly impacts the data quality. Various extraction techniques, ranging from conventional methods like maceration [11] to greener approaches like pressurized liquid extraction (PLE) [12, 13], have been employed. Extraction performance depends on factors such as solvent, temperature, time, and pressure, contributing to variability in interferences and chromatographic quality [14]. While extraction methods are often selected based on the amount (concentrations) of extracted compounds [13], considering their contribution to the overall selectivity towards target compounds is essential, particularly given the diverse chemical composition of berries. Although some of the compounds such as amino acids are relatively low abundancies in berries, their effects on the quantification of target polyphenols are inevitable [15]. In the diode array detector (DAD) such compounds may overlap with the signal of interest, while in the mass spectrometry, they might diminish ionization efficiency.

In chromatography, it is also important to choose a proper sample preparation technique to reduce the complexity of the matrix. An extraction method that introduces fewer interferences in the chromatogram can be desirable, enhancing the purity of peaks. Therefore, evaluating both extraction selectivity and chromatographic resolution is vital for ensuring data quality. In a simple DAD, there also exist methods such as peak purity check which uses peak purity index and peak purity match factors in estimating the interferences [16]. However, the limitation of such methods is that they are not powerful enough to detect impurities existing at low concentrations as demonstrated by Wiberg et al. [16]. In addition, resolving highly overlapping peaks may also be a challenge.

Chemometrics tools such as multivariate curve resolution alternating least squares (MCR-ALS) offer promising solutions as one of the advanced analytical methods to assess extraction selectivity comprehensively. MCR-ALS is one of the methods that unfold bilinear data by employing algorithms such as singular value decomposition (SVD) and principal component analysis (PCA), which enables the resolution of spectral mixtures into individual chemical compounds [17]. The advantage of the MCR algorithm over a simple peak purity check is that it is capable of revealing interferences as well as other small amounts of impurities covered under other peaks [16]. The analyte of interest may be efficiently extracted from other co-eluting components, while uncovering the information about the nature of interfering components. Compared with other approaches which can also be employed in qualitative and quantitative analysis of polyphenols such as mass spectrometry, MCR offers the benefits of non-instrumental needs, low cost, and simplicity. MCR-ALS algorithms have been successfully applied in the resolution of overlapping compounds in chromatography [18, 19] and detection selectivity [20]; however, its application in evaluating extraction performance is underexplored. Only one study by Abrahamson et al. [21] demonstrated that multicurve resolution can be employed in online SFE-UV/VIS where extraction kinetics and overlapping components were successfully determined. Another important feature in MCR-ALS is evolving factor analysis (EFA), which is known for its exploration of degradation components. By carefully studying the chromatographic data set of samples exposed to different storage conditions, EFA can be utilized to establish the emergence and decay of compounds [22]. Studying the information about components that form after degradation could facilitate a scaled-up prediction of degradation of products [22, 23].

In this study, we demonstrate that the chemometrics method MCR-ALS can be used to determine extraction selectivity for different compound classes using simple LC-DAD data. To the best of our knowledge, this is the first time where MCR-ALS is utilized to address the quantification of selectivity in extraction. In addition, we utilize both LC-DAD and MCR-ALS for quantification and assessing the storage stability of polyphenols in berry smoothies. The choice of application scope was motivated by the fact that currently, data on appropriate storage conditions as well as extended storage time for different berry products is scarce, particularly in relation to wild forest berries such as lingonberries and bilberries.

## Materials and methods

### Reagents and standards

Standards of cyanidin, malvidin-3-glucoside, cyanidin 3-O glucoside, epigallocatechin, epicatechin, petunidin 3-O glucoside, pelargonidin 3-0 glucoside, peonidin 3-O glucoside, delphinidin 3-O glucoside, catechin, resveratrol, rutin, kaempferol, and kaempferol 3-O glucoside were purchased from Extrasynthese (Geney, France); myricetin, quercetin, quercetin 3-O glucoside, gallic acid, p-coumaric acid, ferulic acid, caffeic acid, trans-cinnamic acid, vanillic acid, 4-hydroxybenzoic acid, 3,5-dihydroxybenzoic acid, chlorogenic acid, and syringic acid were purchased from Sigma-Aldrich (St Louis, MO, USA). LC-MS grade methanol (MeOH) 99.99%, ethanol (EtOH) 99.7% and formic acid (FA) 98% were all obtained from Sigma-Aldrich (St. Louis, MO, USA). Ultrapure water (18.2 MΩ/cm) was purified by a Milli-Q purification system (Millipore, Billerica, MA, USA), and nitrogen (99.999%) was obtained from AGA industrial gases (Lidingö, Sweden).

Stock solutions of 1000 mg/L were prepared for each standard in methanol. Linear calibration curves were prepared in the range of 2–100 mg/L, except for anthocyanins which were prepared in the range of 2–200 mg/L.

### Samples and sample storage

Frozen bilberries (*Vaccinium myrtillus*) and lingonberries (*Vaccinium vitis-idaea*) were mixed with grape juice (*Vitis vinifera*) into berry smoothie drinks (TetraPak AB & Berry Lab AB, Lund, Sweden). The frozen bilberries and lingonberries were derived from Olle Svensson AB (Olofström, Sweden). Red grape juice (not-from-concentrate) from Julian Soler (S.A. Quintanar del Rey, Cuenca, Spain). For the purpose of this study, products were used and stored for chemical analysis, and the rest was used in vivo and clinical trials. Before analysis, some portions of the berry smoothie were freeze-dried, while the others were kept fresh. The drinks were stored at − 80 °C until further analysis of polyphenol composition. Berry smoothie drinks packaged in Tetra Brik® Aseptic carton material were stored at various temperatures; room temperature (22 ℃), fridge (4 °C), and freezer (− 20 °C), where each time a new box was opened during analysis. The baseline measurement started on the day the berry smoothie was freshly produced. Triplicates and duplicates of berry smoothie stored in the fridge and at room temperature, respectively, were analyzed after 2 weeks, 4 weeks, 3 months, 6 months, 9 months, and 12 months of storage, between May 2020 and May 2021. Triplicates of berry smoothies stored in the freezer were analyzed after 3 months in May 2020, and measurement was continued at 3-month intervals until 18 months and then 6-month intervals until a period of 30 months.

### Sample preparation methods

Extraction conditions were studied by comparing four different protocols common to the analysis of polyphenol compounds in fruits and fruit juices. The protocols are summarized below:

#### Pressurized liquid extraction (PLE)

The method was adopted from Arapitsas and Turner [24]. Briefly, approximately 1 g of freeze-dried sample was weighed. The samples were extracted using a Dionex ASE-350 system (Thermo Fisher, Germering, Germany). The solvent used was water/ethanol/formic acid (94/5/1*v/v/v*) pressurized at 103 bar. The extraction was carried out with two cycles at a temperature of 99 °C with a preheating time of 6 min and extraction time of 10 min.

#### Ultrasonication-assisted extraction (UAE)

The method involved briefly weighing 5 g of fresh sample (1 g was weighed for the freeze-dried sample). Five milliliters of 1% formic acid (FA) in methanol was added to the sample before sonication with an ultrasonic bath for 30 min at 40 °C, and subsequently, the supernatant was collected after centrifugation.

#### Centrifugation

This procedure involved centrifugation only, applied to the fresh berry smoothie sample (5 g). With no solvent added, samples were centrifuged for 15 min at 4 °C, and the supernatant was collected by decanting.

Finally, fresh and freeze-dried samples were compared using UAE, and these were labeled UAE-FS and UAE-DS, respectively. All the extracts were diluted to 25 mL with 1% FA in water prior to HPLC analysis.

### Moisture determination

Moisture content was determined according to the AOAC 2000 [25] method. Briefly, weighing 3 g sample in triplicates, then drying in an oven at 105 °C overnight, and weighing the residue.

### pH determination

The pH was determined using the FiveEasy Plus benchtop FP20 pH/mV meter (Mettler-Toledo AB, Stockholm, Sweden). The pH of the berry smoothie was measured under three different storage conditions: freeze (− 20 ℃), fridge (4 ℃), and room temperature (22 ℃), and the samples were studied in time intervals explained in the “Samples and sample storage” section.

### Analysis by high-performance liquid chromatography diode array detection

The HPLC system was an Agilent HPLC 1100 series, with a diode array detector (DAD) (Agilent Technologies, Waldbronn, Germany). The system also consisted of a G1312A binary pump, a G1329A autosampler, a G1379B degasser, a G1316A thermostat column, and a G1315B photodiode array detector, controlled by ChemStation software (Agilent, v.01.03). Chromatographic separation condition and setting were as follows: column used, Kromasil RP C-18 (150 × 4.6 mm, 3.5 μm); mobile phase, (A) 1% FA in water and (B) 1% FA in acetonitrile; temperature, 40 °C; flow rate, 0.5 mL/min; injection volume, 20 μL. The gradient was set as follows: 0–5 min (2% B), 10 min (7% B), 20 min (8% B), 25 min (10% B), 30 min (10% B), 40 min (16% B), 45 min (25% B), 50 min (50% B), 55 min (95% B), 60 min (100%); equilibration time: 3 min. Detection was done at selected wavelengths for different compound groups, where 280 nm was used for phenolic acids, except for chlorogenic acid, resveratrol, caffeic acid, ferulic acid, and p-coumaric acid (measured at 325), 360 nm for flavonoids, and 520 nm for anthocyanins. Spectral data from all peaks were also recorded in the range 200–700 nm. The injection volume employed was large enough to enable detection of as many phenolic compounds as possible. Similarly, a relatively low flow rate was selected (0.5 mL/min) in order to enhance the detection sensitivity of the DAD.

### Quantification method 1: total peak areas at the selected wavelengths

In most cases, finding all the chemical standards to perform precise quantification is a challenge. Therefore, a classical approach often used is to estimate the total amounts at specific wavelengths (λ), such as 280 nm for phenolics, 360 nm for flavonoids, and 520 nm for anthocyanins. However, this method is typically non-selective and considered an approximation due to other interfering components. Spectral absorption, being an additive signal, means that any compound with a significant molar absorptivity at a particular wavelength will contribute to the overall signal. Therefore, spectral overlaps between different compound classes further complicate the estimation, which requires a close inspection. The classical quantification of total extracted polyphenols was done by using gallic acid equivalents (GAE), quercetin equivalents (QE), and cyanidin 3-0 glucoside equivalents (CE) for total phenolics, total flavonoids, and total anthocyanins, respectively [13, 26, 27]. Individual compounds were tentatively identified by their DAD spectral fingerprint and chromatographic retention time compared with their standards. Average concentrations of polyphenols (mg/100 g) were calculated through external calibration of individual standards. All measurements were done in triplicates (*n* = 3).

### Quantification method 2: HPLC–DAD with MCR-ALS data processing

MCR-ALS is another quantitative and qualitative approach that addresses the challenge of overlaps through spectral deconvolution so that each compound class can be quantified separately. MCR-ALS was done with the MATLAB toolbox [28]. The algorithm decomposes the two-way data matrix into relative concentration profiles and spectra of pure spectra. The two-way data can be described according to Eq. 1 [29]:

1 $$D=C\times {S}^{T}+E$$

where *D* (*N* × *M* $$)$$ is the data matrix containing a mixture of compounds, *C* (*N* × *J*) is the concentration profiles of each pure component, *S* (*M* × *J*) is the spectra of pure components, and *E* (*N* × *M*) represents the error of the matrix. The variables *N*, *M*, and *J* represent the number of chromatographic scans (N), the wavelength range (M), and the number of components (J). The iterative least squares algorithm (ALS) deconvolutes the matrix into C and S by minimizing the error matrix (E). The model can be constrained and iterations are run until the minimum error in the model is achieved. In this particular study, the constraints implemented were non-negativity for both concentration and spectra profiles. Savitzky-Golay smoothing was applied in both spectral and time dimensions using window sizes of 10 and 3, respectively.

Evolving factor analysis was utilized to explore degradation components. The chromatographic data can be concatenated into an augmented matrix, where compounds formed can be explored according to their elution times as demonstrated by Roma et al. [30]. However, concatenating such data can be a challenge for studies done over a long period, especially due to retention time shifts. In the context of our transformation experiments, only a few chromatographic runs (baseline storage to one-month storage) had less retention shifts. To obtain a comprehensive understanding of degradation, the observed spectra in a chromatogram were combined to form a total spectrum, which indicated the overall absorbance of the sample. Data analysis of experiments at different storage temperatures was conducted simultaneously after arranging the obtained spectral data in order, from baseline storage to the final last storage temperature, resulting in a column-wise augmented matrix. Given its powerful resolution, MCR-ALS employing EFA can also explore the full spectral information, instead of exploring the distinct bands in a chromatogram. This information was used to provide useful qualitative information about the evolution of components during storage, without employing reference standards. EFA procedure involves performing a PCA analysis on expanding data matrices, such as spectral observations from a chemical reaction recorded as a function of time. The eigenvalues obtained from each submatrix can be plotted against time to investigate the process trajectories. The PCA is performed from the top of the data set, termed forward EFA, while backward EFA implies that PCA is performed from the bottom to the top of the data set. This method allows for the correlation of forward EFA eigenvalues with forming compounds and backward EFA eigenvalues with decaying compounds [30].

### Relative selectivity of extraction methods

The relative selectivity of each method was calculated as the ratio between peak heights of target polyphenols and interferences identified from chromatograms deconvoluted by the MCR-ALS algorithm. The selectivity ratio is presented in Eq. 2:

2 $$Z =\frac{{\sum H}_{T}}{\sum ({H}_{T}+{H}_{I})}$$

where *Z* is the relative selectivity value and *H*_*T*_ and *H*_*I*_ represent the total peak height of the target compound and interferences, respectively. This ratio defines the extraction method’s ability to extract targeted compounds over the interferences. This equation is deduced from our previous study [31], where the ratio of the normalized amount of analyte to the sum of concentrations of all the other components was considered. However, in this context, ratios of peak heights of components were used to compare the selectivity of different extraction methods and to find out which extraction method gives “cleaner” extracts. In addition, the calculated selectivity in this context is assumed to be relative as the composition of the sample is not wholly known.

**Fig. 1:**
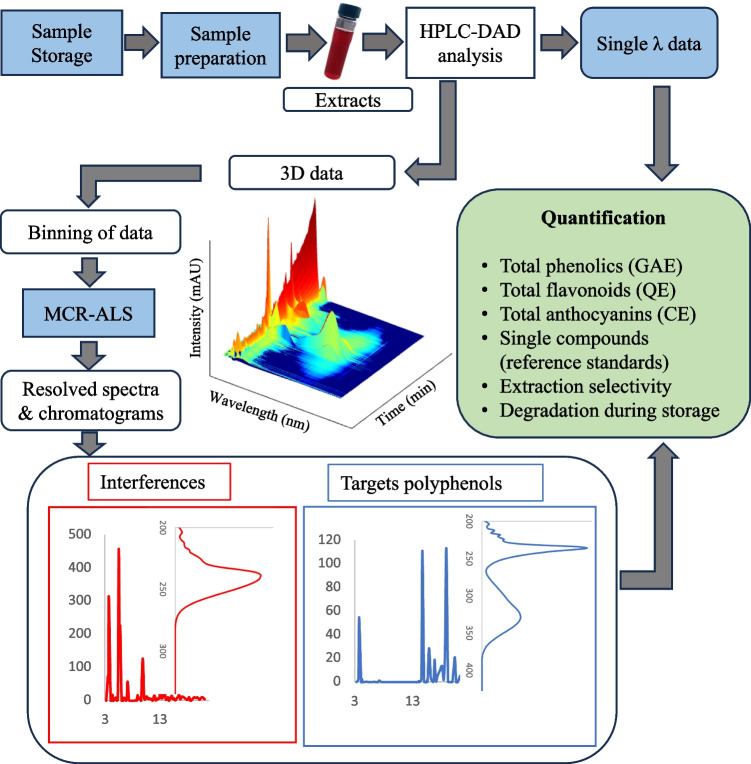
Analyses workflow of the study

Relative selectivity was explored by applying the MCR-ALS algorithm by binning the HPLC-DAD data into different regions of interest (chromatogram shown in Fig. S1, see Electronic Supplementary Material, ESM), i.e., 3–20 min (where most phenolic acids eluted), 20–45 min (where mostly anthocyanins eluted), and finally 45–56 (where flavonoids eluted); see the flow chart in Fig. 1. A standard mixture of 19 polyphenols was also run to confirm the retention times and spectra of target compounds (Table S1 in ESM). In each sample, each MCR component was then associated with a particular compound eluting at a particular retention time. However, only significant eigenvectors explaining the most variability were considered after applying the MCR-ALS algorithm. In general, an extraction method with a high number of spectral overlaps of interfering components other than the desired analyte is deemed to be less selective, which can lead to a high standard error in the prediction of the true concentration of the targeted compounds.

### Degradation kinetic model

The degradations of phenolic compounds were fitted through a first-order model (Eq. 3), whereas the half-life was calculated through Eq. 4.

3 $${C}_{t}=\text{C}0 {e}^{(-k\times t)}$$

4 $${t}_{1/2}=\frac{ln2}{k}$$

*C*_*t*_ refers to the concentration of the compound at the given time *t* (min), and *Co* shows the initial concentration of the compound at time *t* = 0.

*k* is defined as the first order rate constant (month^−1^), while the t_1/2_ refers to the half-life time.

### Statistical analysis

Analysis of variance (ANOVA) was applied to assess the significance of the observed variations in phenolic compound content. ANOVA was applied comparing the significance of the variance between extraction methods as well as the storage period. On the other hand, paired *t*-test statistic was used to compare the significance of the two different methods. The significance of the mean values was considered at *P* < 0.05.

## Results and discussion

### Comparisons of quantification of total polyphenols using LC-DAD single wavelength and MCR-ALS

**Fig. 2:**
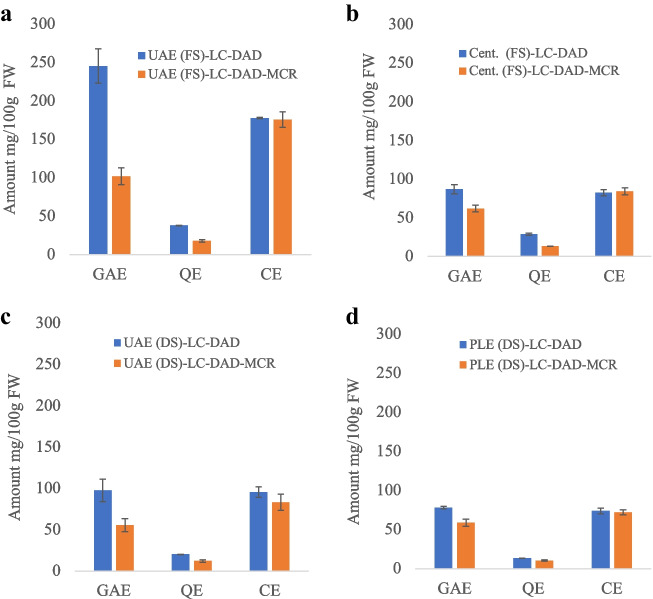
Total phenolics (GAE), total flavonoids (QE), and total anthocyanin (CE) content were extracted by four different extraction procedures and quantified using two different strategies (LC-DAD-single λ and MCR-ALS). Total phenolic compounds extracted by MCR-ALS exclude flavonoids, anthocyanins, and other interfering organic acids. FW represents fresh weight, while DS and FS refer to dried (freeze-dried) samples and fresh samples, respectively. Error bars represent the standard deviation for *n* = 3 extractions

In this study, a comparison of the total amounts of different polyphenol classes (phenolic acids, flavonoids, and anthocyanins) were compared by employing LC-DAD and applying MCR-ALS signal processing. The LC-DAD strategy involves quantification at a single wavelength selected to represent the target compounds, while in MCR-ALS, the whole spectra of the compound are considered after unmixing from other components. However, for quantification, both methods employ a single reference standard. Total phenolics (usually quantified at 280 nm) encompass a diverse group, including phenolic acids, flavonoids anthocyanins, and other compounds. Spectral overlap among these groups complicates estimation, particularly distinguishing between polyphenols like phenolic acids and anthocyanins. To appreciate the significance of the overlaps between compounds of different classes, their molar absorptivity is compared at different wavelengths as shown in Table S2 (see ESM). Compounds with high molar absorptivity such as anthocyanins and flavonoids give a considerable signal at 280 nm; therefore, estimating phenolic acids is almost impossible with single wavelength quantification. Several other interferences such as amino acids and organic acids have been shown to increase the risk of overestimating the phenolic amounts as they also have significant absorptivity at 280 nm (Table S2 in ESM). Therefore, at this wavelength, selectivity is generally lacking. However, after applying the MCR-ALS algorithm, the total phenolic acids were able to be estimated without the influence of anthocyanins, flavonoids, and some small organic acids and amino acids, compared with the amounts estimated by LC-DAD single wavelength as shown in Fig. 2. However, since the identities of the compounds were mainly based on spectral profile, it has to be pointed that it is still uncertain that the amount estimated by MCR-ALS could be all phenolic acids, as other amino acids might exhibit same spectral profile as target compounds. However, the contributions of some small organic acids such as maleic acid, ascorbic acid, and amino acids such as tryptophan were confirmed by their reference standards after MCR-ALS extraction (Fig. S2, see ESM). The initially high estimation of flavonoid contents before MCR-ALS also suggests contributions from compounds other than flavonoids (Fig. 2), which could likely be phenolic acids absorbing at slightly higher wavelengths such as chlorogenic acid, as well as anthocyanins. However, total anthocyanin shows no significant difference between quantification with and without MCR-ALS processing, confirming the selectivity of the high wavelength (520 nm) used (Fig. 2).

More importantly, the significance of this observation varies among extraction methods; for instance, flavonoids extracted by the PLE technique show minimal difference before and after MCR-ALS, indicating fewer interfering components. Conversely, after MCR-ALS, other methods exhibit approximately half the amount of flavonoids recorded by LC-DAD single λ quantification, suggesting more interfering compounds. Interestingly, UAE assisted with acidified methanol as a solvent also indicated a high difference between the total phenolic amounts estimated by LC-DAD-single λ and MCR-ALS compared with other techniques, which might suggest that the method is more comprehensive in extracting several compounds. While this could be reflecting the differences in extractability of components (target compounds and interferences) between extraction conditions of techniques employed, it could also possibly suggest that the nature of interferences varies between these extraction methods. In addition, some variation might be due to the difference between the freeze-dried sample and the fresh samples.

### Comparisons of extractability of total polyphenols between extraction methods

In general, investigating the extractability of three different groups of phenolic compounds (phenolic acids, anthocyanins, and flavonoids), employing the four different extraction protocols indicated that UAE-FS yielded the highest extractability (*P* < 0.05), regardless of the quantification strategy employed (Fig. 2). This increased extractability with MeOH as extraction solvent compared with PLE where the solvent is mostly water may stem from its lower solvent polarity, which facilitate the extraction of a wide range of phenolic compounds. Similar studies have supported the efficacy of water-alcohol mixtures in particular, as the best solvent for flavonoids and anthocyanins compared with water (only) infusions, as demonstrated in lingonberries [32, 33]. Extraction in acidic conditions proved highly favorable for anthocyanins, stabilizing their cation-charged flavylium form [34], as well as improving peak shapes in HPLC analysis. Sample preparation by centrifugation of the aqueous layer from the fresh sample, while advantageous for preserving thermally labile phenolic compounds due to its lack of heating, resulted in the poorest extraction of polyphenols, possibly due to its inability to extract polyphenols bound to the matrix. Conversely, PLE using water/ethanol/FA (95/4/1, v/v/v), under pressurized conditions, exhibited lower extractability of the phenolics, flavonoids, and anthocyanins compared with the acidified methanol with UAE. The elevated temperatures in PLE (100 °C) may decrease the viscosity, aiding solvent penetration of the matrix to access bound phenolic compounds, yet it could also lead to compound degradation. In contrast, milder temperatures (40 °C) employed in UAE may be suitable for balancing extractability and minimizing degradation.

The observed discrepancies could not only be attributed to the effect of solvent mixtures but also other factors such as extraction time, pressure, and temperature which were different between techniques. The difference between fresh and freeze-dried samples also marks a major contribution in the observed variation extractability (compared with the UAE method), where higher extractability was observed in fresh samples compared to freeze-dried samples, among all compounds groups (Fig. 2). To further explore the cause of these difference, an investigation was conducted by adding of the same amount of water content as fresh sample (85.5% moisture) to the freeze-dried samples (Fig. S3, see ESM). The results revealed about 30% increase in anthocyanin content from the re-moisturized freeze-dried sample, compared with the dry berry smoothie sample (Fig. S3, see ESM). However, the addition of water did not alter the extractability of total flavonoids. Nonetheless, it is not possible to conclude if these observations are an indication of losses freeze-during drying or alteration of the sample morphology due to freeze-drying affecting the extractability of compounds. However, loss of anthocyanins during the freeze-drying process has been previously reported [35], linking them to increased enzymatic activities resulting from cell disruption and prolonged freeze-drying time.

### Comparisons of selectivity of different extraction methods

**Fig. 3:**
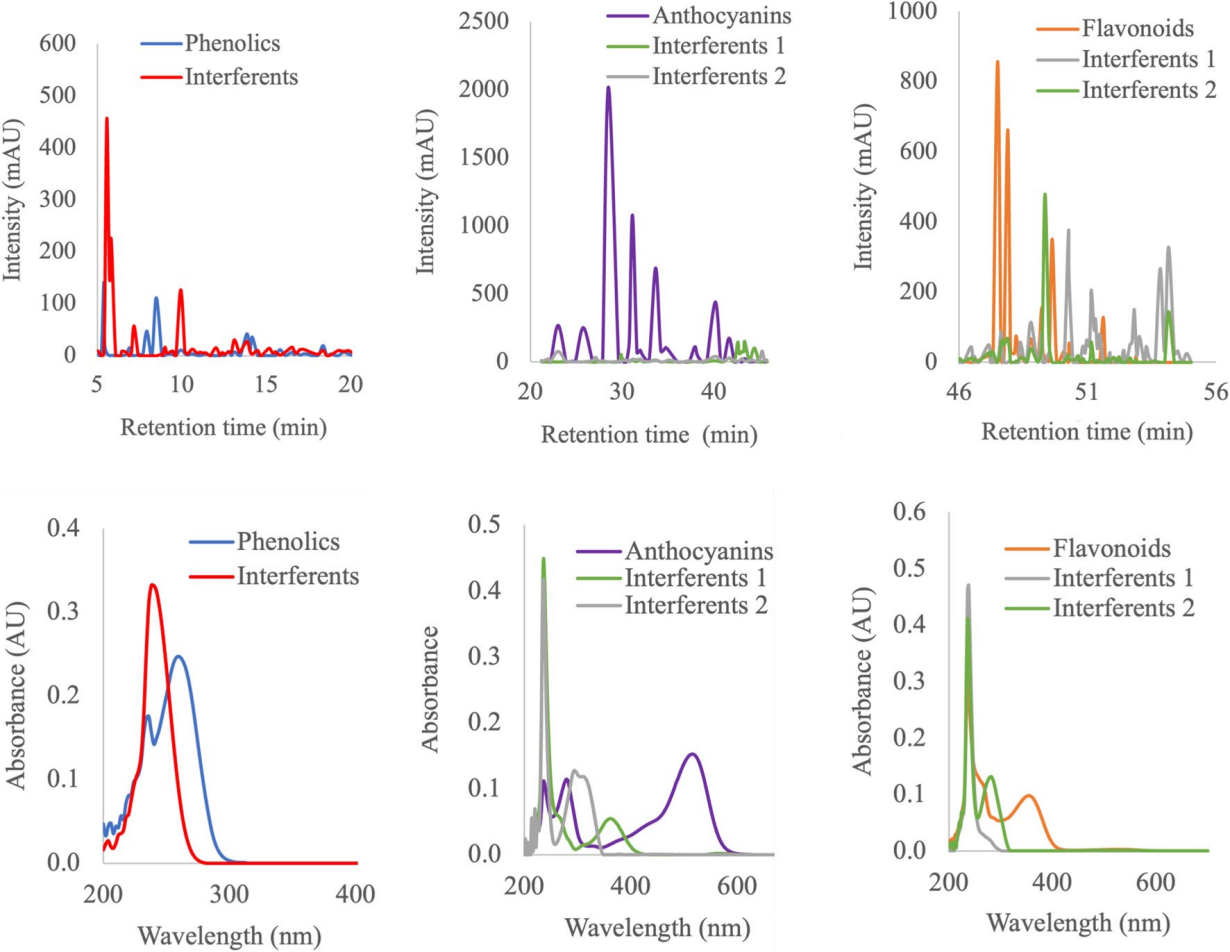
Chromatograms (top) and corresponding spectral profiles (bottom) showing target and overlapping interferences obtained after MCR-ALS analysis of different elution ranges, i.e., 3–20 min (top, left), 20–45 min (middle), and 45–55 min (top, right)

In this study, data sets from different extraction protocols were also subjected to MCR-ALS to evaluate and compare various extraction protocols regarding the significance of interferences relative to target polyphenolic compounds, as indicated in the flow chart in Fig. 1. As already indicated, quantification typically conducted at a single wavelength may be hindered by spectral overlaps from interfering compounds. The precision of quantification also relies on the extraction method’s selectivity, with highly selective methods producing cleaner extracts with fewer spectral overlaps. MCR-ALS was used to estimate the nature of interferents among different extraction methods, given its ability to provide information about the interferent’s spectra. Interferences were evaluated in three chromatographic ranges (3–20, 20–45, and 45–55 min), yielding satisfactory fittings for all extraction methods, with explained variances greater than 99% and lack of fit less than 10%. As shown in Fig. 3, MCR-ALS has shown the ability to enhance chromatographic resolution, which allowed for the investigation of overlapping unknown interfering peaks. More importantly, interfering peaks were observed in the region between 3 and 20 min where most phenolic acids such as gallic acid eluted and 45–55 min where most flavonoids eluted. Overall, the region where anthocyanin eluted (20–45 min), showed very minor interferences. In the 3–20 min chromatogram region, major interfering compounds exhibited absorption spectra around 248 nm (Fig. S2, see ESM), which are likely organic acids, particularly intense in the extract from PLE and centrifugation.

**Fig. 4:**
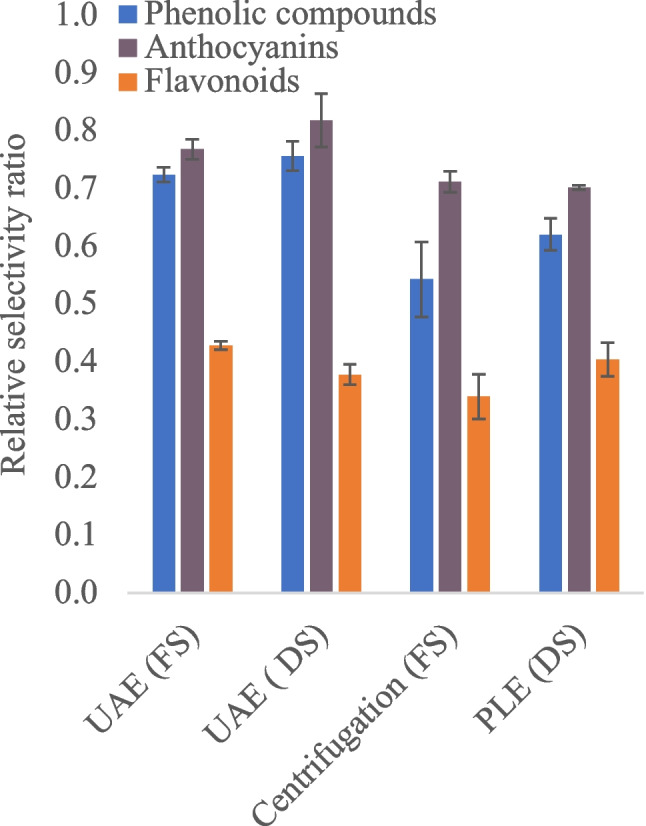
Relative selectivity of different extraction methods on the extraction of phenolic compounds (excluding anthocyanins and flavonoids), anthocyanins and flavonoids, against other interfering compounds as elucidated by MCR-ALS. Error bars represent the standard deviation for *n* = 3 extractions. DS refers to freeze-dried samples, while FS is fresh samples

Relative selectivity values, ranging from 0 to 1 (where 1 represents maximum selectivity), were calculated by comparing confirmed target compounds with interfering peaks (Fig. 4). Results indicated that UAE with acidified MeOH as a solvent exhibited better selectivity on average compared with other extraction protocols, particularly for early eluting compounds such as phenolic acids and anthocyanins (Fig. 4). For instance, for the observed peaks eluting between 3–20 min, we can estimate that 72% of the total observed signal intensity can be attributed to phenolic compounds, while 28% accounts for other interferences when using UAE. Conversely, the PLE technique and centrifugation of the fresh sample showed more overlapping non-target peaks, likely due to water contributing to the extractability of polar compounds like amino acids, an observation in line with the previous study [31]. However, this also suggests that these methods enable the high extractability of these small untargeted organic acids, compared with UAE with methanol. Limited selectivity for flavonoids eluting in the 40–45 min region was observed across all extraction techniques, likely due to the ability to extract non-polar organic compounds like terpenes. It should be stated that some of the major overlapping compounds in the region where flavonoids eluted were phenolic acids such as p-coumaric acid, which were not considered as interferences for the calculation of extraction selectivity since they are part of the target compounds when considering the whole spectrum. Although elucidation of all possible interfering peaks might be a challenging task and a limitation in this study, a screening with high-resolution mass spectrometry and MS DIAL library search helped to annotate both target compounds (Table S1, see ESM) and few interferents (Table S3, see ESM). The details of mass spectrometry experiments are shared in S1. As shown in Fig. S4 in the ESM, the potential interferents were plotted in a scatter plot to visualize how they are retained relative to different groups of analytes of interest (phenolic acids, flavonoids, and anthocyanins) on a C18 column. It is quite clear that most of the early eluting peaks are small organic acids such as ascorbic acid, quinic acid, and maleic acid. In addition, some of these interferences were further confirmed by the use of standards in HPLC, and the peaks are marked in Fig. S2 in the ESM. These organic acids are quite common in berry fruits [36, 37]. Other interferences eluting in the region where most of the target analytes eluted were possibly various heterocyclic compounds and terpenes (Fig. S4, see ESM). While these interferences might not all have a significant impact on the quantification of anthocyanins and flavonoids as they absorb light at fairly high wavelengths, their contribution to the analysis of other compounds like phenolic acids could be impactful, as shown by the molar absorptivity data in Table S1 in the ESM.

### Evaluating the effect of overlapping peaks on single compound quantification

Further characterization was carried out by identification and quantification of individual peaks forming the phenolics, flavonoids, and anthocyanins (annotated in Fig. S1, see ESM). The method of choice employed for extraction was UAE (fresh sample) with acidified MeOH, as extraction solvent, as it exhibited the highest extractability and fair selectivity among all the compound groups. It can be noted that the majority of peaks detected from 26 to 60 min were anthocyanins which also show a signal at 280 nm (Fig S1, see ESM). Nineteen compounds were confirmed by their retention times, UV spectra, and mass spectra in the berry smoothie samples, of which 7 compounds belong to the anthocyanin group, 7 flavonoids, 7 phenolic acid and 1 stilbene (Table S2, see ESM). Of these detected compounds, 15 compounds were quantified relative to their reference standards as shown in Table 1. It was observed that 3-O glycosylated anthocyanins were detected in higher amounts followed by chlorogenic acid. Several findings reported cyanidin, delphinidin, pelargonidin, peonidin, malvidin, and petunidin, in their glycosylated form, as the most anthocyanidins distributed in the various berries [38, 39]. Phenolic acids content were generally in low amounts (some even below the limit of quantification), and only chlorogenic acid was found in substantial amounts. Chlorogenic acid has been also identified as the main phenolic acid in blueberries [40].

**Table 1:** Amounts of single compounds after quantification with both LC-DAD and LC-DAD with MCR-ALS data processing, in berry smoothie sample extracted with UAE using 1% FA in methanol. Paired *t*-test was used to compare the significance of the variability of quantification between the two methods employed (*p*-value)

Compound name	Amounts (mg/100 g of fresh weight sample)		*p*-value
	LC-DAD	LC-DAD-MCR-ALS	
Delphinidin 3-0 glucoside	18.4 ± 1.7	20.4 ± 0.5	0.14
Cyanidin 3-0 glucoside	34.8 ± 3.5	34.3 ± 0.8	0.22
Petunidin 3-0 glucoside	13.6 ± 0.3	13.9 ± 0.2	0.90
Pelargonidin 3-0 glucoside	6.2 ± 0.5	6.5 ± 0.2	0.74
Peonidin 3-O glucoside	11.9 ± 0.5	11 ± 0.1	0.64
Malvidin 3-O glucoside	11.6 ± 0.3	10.3 ± 0.5	0.13
Rutin	6.4 ± 0.1	5.5 ± 0.38	0.07
Quercetin-3-0-glucoside	4.3 ± 0.03	2.9 ± 0.3	0.01
Kaempferol-3-0-glucoside	2.7 ± 0.09	3.0 ± 0.2	0.17
Mycetrin	1.3 ± 0.02	1.2 ± 0.1	0.25
Quercetin	1.2 ± 0.1	1 ± 0.06	0.10
Trans-Cinnamic acid	0.5 ± 0.03	0.5 ± 0.03	0.14
Gallic acid	0.8 ± 0.05	0.9 ± 0.05	0.67
Chlorogenic acid	9.9 ± 0.03	7.6 ± 0.4	0.08
Resveratrol	0.6 ± 0.01	0.48 ± 0.06	0.12
Total phenolics (280 nm)	245.4 ± 22.4	101.9 ± 10.9	0.007
Total flavonoids (360 nm)	37.9 ± 0.4	17.9 ± 1.9	0.001
Total anthocyanins (520 nm)	177.8 ± 1.0	175.6 ± 10.1	0.60

However, to establish how much the overlapping interferences affected the quantification of the detected compounds, the quantification of single compounds using both HPLC-DAD and MCR-ALS processing was compared (Table 1). Since the true concentration in the sample is unknown, differences between the methods may indicate impurities or variability due to the quantification methods used. However, we can assume that an agreement between the two methods confirms the purity of the peak as well as the quality of the chromatographic separation, given the ability of MCR-ALS to detect impurities covered under the peaks. Overall, most peaks indicated valid purity, showing *p*-values above 0.05 when comparing quantification with LC-DAD and MCR-ALS. This was particularly evident for the anthocyanins, where high selectivity is expected even with single wavelength quantification. Interestingly, flavonoids, which exhibited numerous interferences among all the extraction techniques, also showed less difference between individual quantified peaks with both LC-DAD and MCR-ALS, except for quercetin 3-O glucoside which indicated slightly higher variability. Despite several interferences observed in the region where flavonoids eluted, closer evaluation indicates that most of the interferences did not co-elute with the identified target compounds. These also approve the contribution of chromatographic resolution considering that these compounds eluted late (45–60 min) allowing for better resolution due to increased retention. In addition, other interferences showed very low absorptivity at 360 nm, rendering the quantification at that wavelength selective.

### Evaluating the long-term stability of polyphenols using LC-DAD and EFA

The developed method was utilized to study the degradation of polyphenols quantified in Table 1. However, the stability study was focused on flavonoids and anthocyanins only, as the number of phenolic acids identified was limited and their concentrations were low. The berry smoothie samples were extracted with UAE with 1% FA in MeOH. LC-DAD was utilized for quantification, while MCR-ALS was the supporting tool used to survey interfering peaks. EFA, in particular, was utilized to elucidate the degradation products, without the use of reference standards. The findings were also used to model the degradation kinetics of polyphenols following storage at various temperatures.

**Fig. 5:**
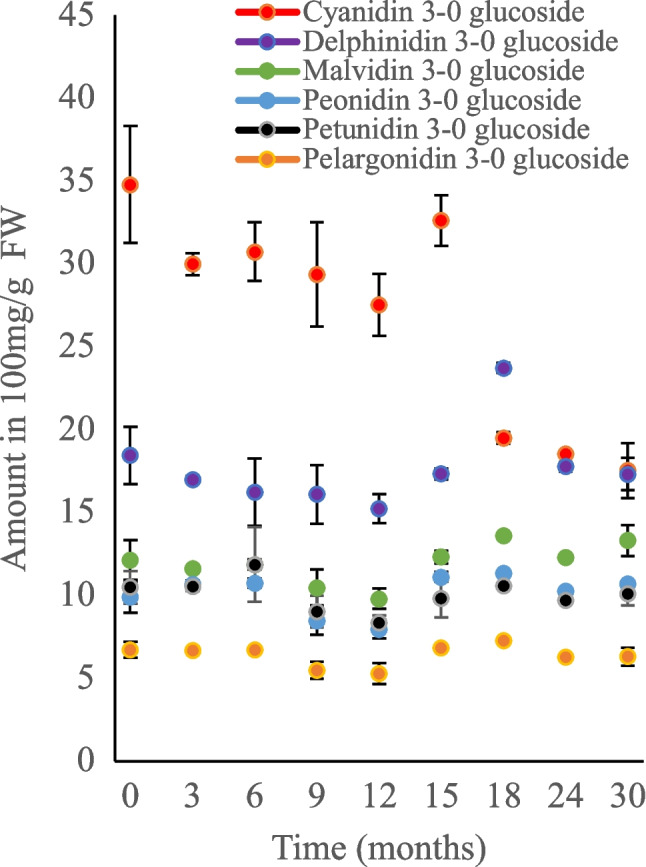
Comparison of anthocyanins response over 30 months in berry smoothie stored in freezer (− 20 °C). Error bars represent the standard deviations for *n* = 3 samples extracted

**Fig. 6:**
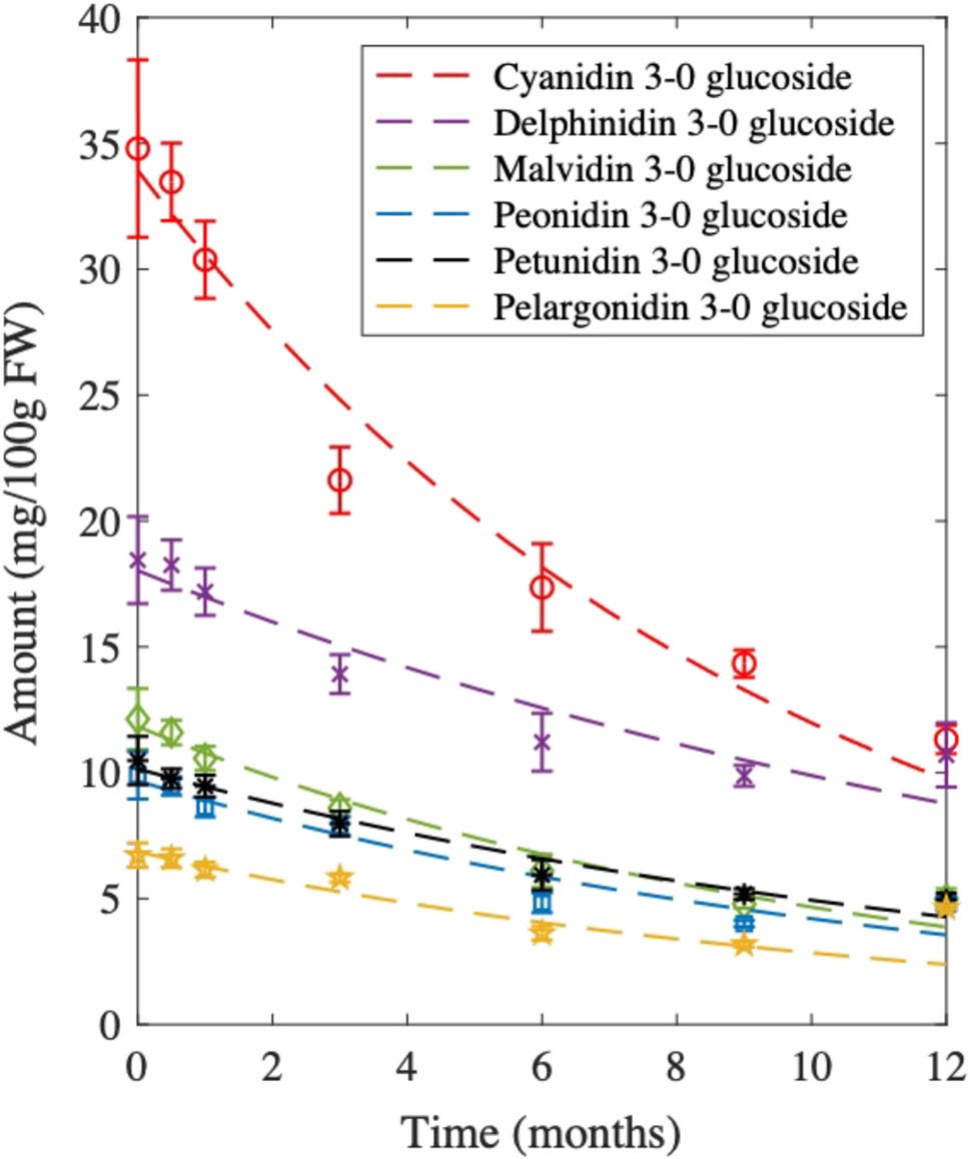
Comparison of anthocyanins response over 12 months of berry smoothie stored at room temperature (fitted with first-order kinetic model). Error bars represent the standard deviations for duplicate samples

In general, anthocyanins showed the most variability as a function of temperatures compared with flavonoids. Most anthocyanins maintained stable concentration for almost 18 and 12 months in the freezer (Fig. 5) and fridge (Fig. S5, see ESM), respectively, mostly showing insignificance differences in concentration over the studied time (*p* < 0.05). In fact, the degradation of anthocyanins and flavonoids in berry smoothies stored in the fridge and freezer did not fit according to the first-order kinetic model (Eq. 3), except for cyanidin 3-O glucoside (Fig. S6, in ESM), which showed an average half-life of 28 months for both fridge and freezer storage (Table S4, see ESM). However, the degradation of anthocyanins in berry smoothies at room temperature followed the first-order kinetic model (Fig. 6), with individual anthocyanins exhibiting half-lives ranging from 7 to 12 months (Table S4, ESM). The results of the fitting coefficients such as rate constant, coefficient of determination, and root mean square error are shown in Table S4 in the ESM. The anthocyanins storage at room temperature also showed similar degradation rates as reported by Muche et al. [41], for anthocyanins studied in grapes. However, fridge and freezer storage slowed down anthocyanin degradation, approximately four times better than room-temperature storage. Low temperatures reduce the activities of enzymes in the plant cells, particularly polyphenol oxidases and peroxidases responsible for phenolic degradation [42, 43]. Moreover, it is also reported that temperature may influence the equilibrium state of anthocyanins, favoring the chalcone form [44]. Flavonoid contents, as well as the pH of the berry smoothie, were among the variables that remained stable for at least 12 months period across all storage temperatures (Figure S7–10, see ESM). However, a notable drop was observed after 9 months of room temperature and fridge storage for some of the glycosylated flavonoids, probably degrading to their aglycone forms.

**Fig. 7:**
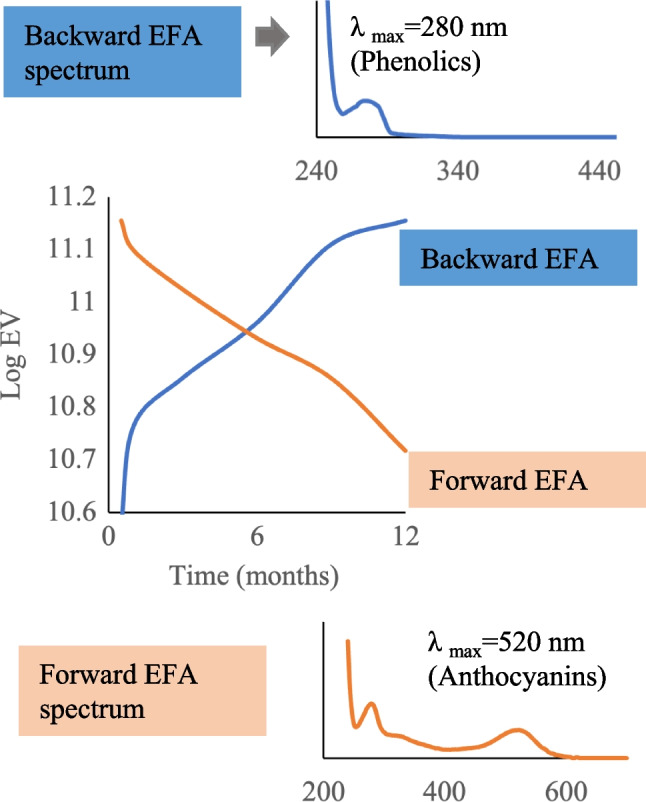
Eigenvector plot derived from EFA, where the orange line shows the forward EFA and the blue line shows the backward EFA, spectral corresponding to anthocyanins and other phenolic derivatives, respectively

The data from room temperature storage samples, where most degradation was observed, was subjected to EFA, to elucidate potential degradation products. From this data set, the singular value decomposition computed five eigenvectors as the representation of the variation in the data. However, only three values were associated with chemical meaning, which was attributed to anthocyanins, other phenolic compounds, and intermediates formed during the process, while the other values were associated with noise in the data. The resultant model led to a lack of fit and explained variance of 0.96 and 99.99%, respectively. The results of EFA are shown in Fig. 7, where the forward EFA plot and the backward EFA plot are overlaid. The scale on the *y*-axis represents the log EV (eigenvalues) of each PCA analysis versus the storage time (*x*-axis). Therefore, the lines connecting the homologous eigenvalues were found to depict the spectra of anthocyanins (shown by forward EFA) and the formation compounds likely to be phenolic derivatives (shown by backward EFA). This also attests to the reports that suggest the degradation of anthocyanins through the hydrolysis of the glycosidic bond connecting the aglycone and glycosyl group, ultimately leading to the formation of products like aldehydes and benzoic acid derivatives [45, 46].

The trend obtained with EFA corresponds well with the degradation of anthocyanins as determined by LC-DAD. Clearly, the break-even point between the degradations and formation of phenolics occurs at around 6 months, which is similar to the average half-life of anthocyanins predicted by first-order kinetics. EFA has been applied previously to predict the kinetic model of bioactive compounds exposed to various conditions [47]. In our study, we correlated the EFA prediction in parallel with utilizing reference standards. By performing EFA on augmented chromatograms from only baseline to 1 months of storage, new peaks of compounds, suspected to be organic acids, were observed, with lambda max around 248 nm (Fig S11, see ESM). These could also suggest that the other compounds break down into small organic acids during room temperature storage of the berry smoothie. The results obtained with EFA were also confirmed by inspecting MCR-ALS profiles from deconvoluted chromatographic data for each storage temperature. The finding also suggests spectral profiles and peaks of some phenolic compounds, found primarily in samples stored for 6–12 months at room temperature (Fig. S12, see ESM), indicating the formation of these compounds after degradation. These outcomes provide insights into the evolution of species during these kinetic processes, especially where no standards are available.

## Conclusions

In this study, we gained valuable insights in handling chromatographic data to improve the quantitative analysis of phenolic compounds in berry smoothies. MCR-ALS was used as a diagnostic tool to assess the selectivity of extraction methods, interferences, quality of chromatographic resolution, and evolution of the degradation process in berry smoothies. The outcomes indicate the utility of this procedure in the estimation of the total concentration of different compound classes, without the effect of other overlapping compounds. By taking advantage of this ability to deconvolute the net signal of LC-DAD data into distinct eluting components, the selectivity of each extraction method was determined by assessing the overlapping components. The outcomes revealed that using acidified methanol as a solvent in UAE on fresh berry smoothie, resulted in the highest extractability of polyphenols, with less amount of interferences, particularly concerning phenolic acids and anthocyanins. PLE and centrifugation showed lower selectivity values, particularly in the region where phenolic acids eluted, possibly due to small organic acids and other polar interferences.

This study also shows the importance of storage temperature for berry products, where room temperature should be avoided as it facilitates the degradation of anthocyanins more than the fridge and freezer storage. The degradation kinetics of anthocyanins at room temperature followed the first-order kinetic model, showing average half of 7 months for anthocyanins during room temperature storage. On the other hand, most anthocyanins maintained at least 12 months of consistent stability in fridge and freezer storage. However, flavonoids mostly retained stability over at least 9 months across all storage temperatures. Although the degradation mechanism of anthocyanins is elusive, the application of MCR-ALS and EFA methods in studying the stability of anthocyanins during sample storage indicated that their degradation possibly leads to the formation of other phenolic-related compounds.

Although the chemometrics tools MCR-ALS demonstrated the potential of quantifying extraction selectivity, one limitation is that not all interferences in the extract can be detected with a UV detector. Thus, the use of several detection techniques could increase the number of interferent compounds detected, yielding a more precise estimation of extraction selectivity. However, MCR-ALS has proved to be a relatively simple analytical tool that can be employed in evaluating performance of extraction and improving the reliability of quantification, without needing several standards to explain many components in the sample.

## Supplementary Information

Below is the link to the electronic supplementary material.

Supplementary file1 (PDF 1.84 MB)

## References

[1] Lock K, Pomerleau J, Causer L, Altmann DR, McKee M. The global burden of disease attributable to low consumption of fruit and vegetables: implications for the global strategy on diet. Bull World Health Organ 2005;83:100–108. /S0042-96862005000200010. PMC2623811 15744402

[2] Hossain MZ, Shea E, Daneshtalab M, Weber JT. Chemical analysis of extracts from newfoundland berries and potential neuroprotective effects. Antioxidants 2016;5. 10.3390/antiox5040036. 10.3390/antiox5040036 PMC5187534 27775557

[3] Marhuenda J, Alemán MD, Gironés-Vilaplana A, Pérez A, Caravaca G, Figueroa F, Mulero J, Zafrilla P. Phenolic composition, antioxidant activity, and in vitro availability of four different berries. J Chem. 2016;2016. 10.1155/2016/5194901.

[4] Koponen JM, Happonen AM, Mattila PH, Törrönen AR. Contents of anthocyanins and ellagitannins in selected foods consumed in Finland. J Agric Food Chem. 2007;55:1612–9. 10.1021/jf062897a. 17261015 10.1021/jf062897a

[5] Heyman-Lindén L, Kotowska D, Sand E, Bjursell M, Plaza M, Turner C, Holm C, Fa F, Berger K. Lingonberries alter the gut microbiota and prevent low-grade inflammation in high-fat diet fed mice. Food Nutr Res. 2016;60:1–14. 10.3402/fnr.v60.29993. 10.3402/fnr.v60.29993 PMC4850145 27125264

[6] Huang F, Marungruang N, Martinsson I, Camprubí Ferrer L, Nguyen TD, Gondo TF, Karlsson EN, Deierborg T, Öste R, Heyman-Lindén L. A mixture of Nordic berries improves cognitive function, metabolic function and alters the gut microbiota in C57Bl/6J male mice. Front Nutr. 2023;10:1–17. 10.3389/fnut.2023.1257472. 10.3389/fnut.2023.1257472 PMC10580983 37854349

[7] Al Hamimi S, Heyman-Lindén L, Plaza M, Turner C, Berger K, Spégel P. Alterations in the plasma metabolite profile associated with improved hepatic function and glycemia in mice fed lingonberry supplemented high-fat diets. Mol Nutr Food Res. 2017;61:1–10. 10.1002/mnfr.201600442. 10.1002/mnfr.201600442 27739180

[8] Marungruang N, Kovalenko T, Osadchenko I, Voss U, Huang F, Burleigh S, Ushakova G, Skibo G, Nyman M, Prykhodko O, Hållenius FF. Lingonberries and their two separated fractions differently alter the gut microbiota, improve metabolic functions, reduce gut inflammatory properties, and improve brain function in ApoE−/− mice fed high-fat diet. Nutr Neurosci. 2020;23:600–12. 10.1080/1028415X.2018.1536423. 30353787 10.1080/1028415X.2018.1536423

[9] Halahlah A, Räikkönen H, Piironen V, Valoppi F, Mikkonen KS, Ho TM. Wood hemicelluloses as sustainable wall materials to protect bioactive compounds during spray drying of bilberries. Powder Technol. 2023;415. 10.1016/j.powtec.2022.118148.

[10] Luís Â, Duarte AP, Pereira L, Domingues F. Interactions between the major bioactive polyphenols of berries: effects on antioxidant properties. Eur Food Res Technol. 2018;244:175–85. 10.1007/s00217-017-2948-5.

[11] Marzullo L, Ochkur O, Orlandini S, Renai L, Gotti R, Koshovyi O, Furlanetto S, Del Bubba M. Quality by design in optimizing the extraction of (poly)phenolic compounds from Vaccinium myrtillus berries. J Chromatogr A. 2022;1677. 10.1016/j.chroma.2022.463329. 10.1016/j.chroma.2022.463329 35863094

[12] Plaza M, Oliveira D, Nilsson A, Turner C. Green and efficient extraction method to determine polyphenols in cocoa and cocoa products. Food Anal Methods. 2017;10:2677–91. 10.1007/s12161-017-0830-5.

[13] Tripodo G, Ibáñez E, Cifuentes A, Gilbert-López B, Fanali C. Optimization of pressurized liquid extraction by response surface methodology of Goji berry (Lycium barbarum L.) phenolic bioactive compounds. Electrophoresis. 2018;39:1673–82. 10.1002/elps.201700448. 29314152 10.1002/elps.201700448

[14] Lefebvre T, Destandau E, Lesellier E. Selective extraction of bioactive compounds from plants using recent extraction techniques: a review. J Chromatogr A. 2021;1635: 461770. 10.1016/j.chroma.2020.461770. 33310280 10.1016/j.chroma.2020.461770

[15] Bastola KP, Guragain YN, Bhadriraju V, Vadlani PV. Evaluation of standards and interfering compounds in the determination of phenolics by Folin-Ciocalteu assay method for effective bioprocessing of biomass. Am J Anal Chem. 2017;08:416–31. 10.4236/ajac.2017.86032.

[16] Wiberg K, Andersson M, Hagman A, Jacobsson SP. Peak purity determination with principal component analysis of high-performance liquid chromatography – diode array detection data. 2004;1029:13–20. 10.1016/j.chroma.2003.12.052. 10.1016/j.chroma.2003.12.052 15032344

[17] De Juan A, Jaumot J, Tauler R. Multivariate curve resolution (MCR). Solving the mixture analysis problem. Anal Methods. 2014;6:4964–76. 10.1039/c4ay00571f.

[18] Sheikholeslami MN, Vosough M, Esfahani HM. On the performance of multivariate curve resolution to resolve highly complex liquid chromatography–full scan mass spectrometry data for quantification of selected immunosuppressants in blood and water samples. Microchem J. 2020;152: 104298. 10.1016/j.microc.2019.104298.

[19] Vecchietti D, Nishio A, Fujita Y, Yoshida T, Yanagisawa T, Kou D. Liquid chromatography coupled with photodiode array and a multivariate curve resolution – alternating least square algorithm for identification and quantification of co-eluting impurities in pharmaceutical analysis. J Chromatogr A. 2022;1678: 463364. 10.1016/j.chroma.2022.463364. 35914409 10.1016/j.chroma.2022.463364

[20] Faber NM, Ferré J, Boqué R, Kalivas JH. Quantifying selectivity in spectrophotometric multicomponent analysis. TrAC - Trends Anal Chem. 2003;22:352–61. 10.1016/S0165-9936(03)00604-6.

[21] Abrahamsson V, Jumaah F, Turner C. Continuous multicomponent quantification during supercritical fluid extraction applied to microalgae using in-line UV/Vis absorption spectroscopy and on-line evaporative light scattering detection. J Supercrit Fluids. 2018;131:157–65. 10.1016/j.supflu.2017.09.014.

[22] Schulz L, Stähle P, Reining S, Sawall M, Kockmann N, Röder T. Multivariate curve resolution for kinetic modeling and scale-up prediction. J Flow Chem. 2023;13:13–9. 10.1007/s41981-022-00252-y.

[23] González-Sáiz JM, Esteban-Díez I, Rodríguez-Tecedor S, Pizarro C. Valorization of onion waste and by-products: MCR-ALS applied to reveal the compositional profiles of alcoholic fermentations of onion juice monitored by near-infrared spectroscopy. Biotechnol Bioeng. 2008;101:776–87. 10.1002/bit.21939. 18814297 10.1002/bit.21939

[24] Arapitsas P, Turner C. Pressurized solvent extraction and monolithic column-HPLC/DAD analysis of anthocyanins in red cabbage. Talanta. 2008;74:1218–23. 10.1016/j.talanta.2007.08.029. 18371772 10.1016/j.talanta.2007.08.029

[25] The Association of Official Analytical Chemists. Official methods of analysis of AOAC International, 17th Editi. MD, USA; 2000.

[26] Meng JF, Xu TF, Qin MY, Zhuang XF, Fang YL, Zhang ZW. Phenolic characterization of young wines made from spine grape (Vitis davidii Foex) grown in Chongyi County (China). Food Res Int. 2012;49:664–71. 10.1016/j.foodres.2012.09.013.

[27] Avram AM, Morin P, Brownmiller C, Howard LR, Sengupta A, Wickramasinghe SR. Concentrations of polyphenols from blueberry pomace extract using nanofiltration. Food Bioprod Process. 2017;106:91–101. 10.1016/j.fbp.2017.07.006.

[28] Jaumot J, de Juan A, Tauler R. MCR-ALS GUI 2.0: new features and applications. Chemom Intell Lab Syst. 2015;140:1–12. 10.1016/j.chemolab.2014.10.003.

[29] de Juan A, Tauler R. Multivariate curve resolution: 50 years addressing the mixture analysis problem – a review. Anal Chim Acta. 2021;1145:59–78. 10.1016/j.aca.2020.10.051. 33453882 10.1016/j.aca.2020.10.051

[30] de Juan A, Tauler R. Factor analysis of hyphenated chromatographic data. Exploration, resolution and quantification of multicomponent systems. J Chromatogr A. 2007;1158:184–95. 10.1016/j.chroma.2007.05.045. 17543980 10.1016/j.chroma.2007.05.045

[31] Gondo TF, Jönsson M, Karlsson EN, Sandahl M, Turner C. Extractability, selectivity, and comprehensiveness in supercritical fluid extraction of seaweed using ternary mixtures of carbon dioxide, ethanol, and water. J Chromatogr A. 2023;1706. 10.1016/j.chroma.2023.464267. 10.1016/j.chroma.2023.464267 37572535

[32] Dewi SR, Stevens LA, Pearson AE, Ferrari R, Irvine DJ, Binner ER. Investigating the role of solvent type and microwave selective heating on the extraction of phenolic compounds from cacao (Theobroma cacao L.) pod husk. Food Bioprod Process. 2022;134:210–22. 10.1016/j.fbp.2022.05.011.

[33] Dróżdż P, Šėžienė V, Pyrzynska K. Phytochemical properties and antioxidant activities of extracts from wild blueberries and lingonberries. Plant Foods Hum Nutr. 2017;72:360–4. 10.1007/s11130-017-0640-3. 29134464 10.1007/s11130-017-0640-3 PMC5717128

[34] Khoo HE, Azlan A, SouTeng Tang SML. Anthocyanidins and anthocyanins: colored pigments as food, pharmaceutical ingredients, and the potential health benefits. Food Nutr Res. 2017;61:1–21. 10.1080/16546628.2017.1361779 PMC5613902 28970777

[35] Seke F, Manhivi VE, Shoko T, Slabbert RM, Sultanbawa Y, Sivakumar D. Effect of freeze drying and simulated gastrointestinal digestion on phenolic metabolites and antioxidant property of the natal plum (Carissa macrocarpa). Foods 2021;10. 10.3390/foods10061420. 10.3390/foods10061420 PMC8235007 34207411

[36] Eyéghé-Bickong HA, Alexandersson EO, Gouws LM, Young PR, Vivier MA. Optimisation of an HPLC method for the simultaneous quantification of the major sugars and organic acids in grapevine berries. J Chromatogr B Anal Technol Biomed Life Sci. 2012;885–886:43–9. 10.1016/j.jchromb.2011.12.011. 10.1016/j.jchromb.2011.12.011 22265666

[37] Gao X, Zhang Z, Wang X, Qian J, Hu L, Li Z, Li W. Studies of value in use, chemical compositions, biological and pharmacological activities, and quality control of Rubus berries: a comprehensive review. J Food Compos Anal. 2023;124: 105707. 10.1016/j.jfca.2023.105707.

[38] Diaconeasa Z, Iuhas CI, Ayvaz H, Rugină D, Stanilă A, Dulf F, Bunea A, Socaci SA, Socaciu C, Pintea A. Phytochemical characterization of commercial processed blueberry, blackberry, blackcurrant, cranberry, and raspberry and their antioxidant activity. Antioxidants 2019;8. 10.3390/antiox8110540. 10.3390/antiox8110540 PMC6912725 31717652

[39] Higbee J, Brownmiller C, Solverson P, Howard L, Carbonero F. Polyphenolic profiles of a variety of wild berries from the Pacific Northwest region of North America. Curr Res Food Sci. 2023;7: 100564. 10.1016/j.crfs.2023.100564. 37664004 10.1016/j.crfs.2023.100564 PMC10474376

[40] Becker Pertuzatti P, Teixeira Barcia M, Gómez-Alonso S, Teixeira Godoy H, Hermosin-Gutierrez I. Phenolics profiling by HPLC-DAD-ESI-MSn aided by principal component analysis to classify rabbiteye and highbush blueberries. Food Chem. 2021;340: 127958. 10.1016/j.foodchem.2020.127958. 32916406 10.1016/j.foodchem.2020.127958

[41] Muche BM, Speers RA, Rupasinghe HPV. Storage temperature impacts on anthocyanins degradation, color changes and haze development in juice of “Merlot” and “Ruby” grapes (Vitis vinifera). Front Nutr. 2018;5:1–9. 10.3389/fnut.2018.00100. 30410884 10.3389/fnut.2018.00100 PMC6209682

[42] Tomás-Barberán FA, Espín JC. Phenolic compounds and related enzymes as determinants of quality in fruits and vegetables. J Sci Food Agric. 2001;81:853–76. 10.1002/jsfa.885.

[43] Araji S, Grammer TA, Gertzen R, Anderson SD, Mikulic-Petkovsek M, Veberic R, Phu ML, Solar A, Leslie CA, Dandekar AM, Escobar MA. Novel roles for the polyphenol oxidase enzyme in secondary metabolism and the regulation of cell death in walnut. Plant Physiol. 2014;164:1191–203. 10.1104/pp.113.228593. 24449710 10.1104/pp.113.228593 PMC3938613

[44] Brouillard R, Delaporte B. Chemistry of Anthocyanin Pigments. 2.1 Kinetic and thermodynamic study of proton transfer, hydration, and tautomeric reactions of malvidin 3-glucoside. J Am Chem Soc. 1977;99:8461–8. 10.1021/ja00468a015.

[45] Weber F, Larsen LR. Influence of fruit juice processing on anthocyanin stability. Food Res Int. 2017;100:354–65. 10.1016/j.foodres.2017.06.033. 28964358 10.1016/j.foodres.2017.06.033

[46] Neuenfeldt NH, de Moraes DP, de Deus C, Barcia MT, de Menezes CR. Blueberry phenolic composition and improved stability by microencapsulation. Food Bioprocess Technol. 2022;15:750–67. 10.1007/s11947-021-02749-1.

[47] Zheng X, Gong X, Li Q, Qu H. Application of multivariate curve resolution method in the quantitative monitoring transformation of salvianolic acid A using online UV spectroscopy and mass spectroscopy. Ind Eng Chem Res. 2012;51:3238–45. 10.1021/ie201536y.

