# Supplementary material for: Investigating the quality of extraction and quantification of bioactive compounds in berries through liquid chromatography and multivariate curve resolution
Source: Anal Bioanal Chem. 2024 Aug 15;416(24):5387–400. doi: 10.1007/s00216-024-05474-8 (PMC11416369; doi:10.1007/s00216-024-05474-8)
Supplement: Supplementary file 1 — Supplementary file1 (PDF 1.84 MB) [file 216_2024_5474_MOESM1_ESM.pdf]

Electronic suplementary material

Investigating the quality of extraction and quantification of bioactive compounds in berries through liquid chromatography and multivariate curve resolution

Thamani Freedom Gondo[a], Fang Huang[b,d], Nittaya Marungruang[c], Lovisa Heyman-Lindén[c,e], Charlotta Turner[a]

[a] Lund University, Department of Chemistry, Centre for Analysis and Synthesis, P.O. Box 124, 22100 Lund, Sweden.

[b] Lund University, Department of Chemistry, Division of Biotechnology, Sweden

[c] Berry Lab AB, Lund, Sweden

[d] Aventure AB group, Lund, Sweden

[e] Lund University, Department of Experimental Medical Science, Lund, Sweden.

*Corresponding author: +46 46 222 8125. Charlotta.Turner@chem.lu.se. https://orcid.org/0000-0001-9466-1149

List of tables

Table S1:Confirmation of identified analytes in berry smoothie samples using high resolution mass spectrometry (HRMS). 4

Table S2: Molar absorptivity of different compounds measured at different wavelengths. 6

Table S3: Interfering compounds of identified analytes in berry smoothie samples using high resolution mass spectrometry (HRMS). 7

Table S4: Kinetic parameters for degradation of anthocyanins derived from first order fitting model. 9

List of figures

Fig. S1: Chromatogram of berry smoothie sample at three different wavelengths. 10

Fig. S2: Interfering organic acids and amino acids identified with standards after HPLC-DAD and MCR -ALS data processing 11

Fig. S3: Total phenolic, total flavonoids and total anthocyanin content extracted using UAE with acidified MEOH, both original fresh sample and freeze-dried sample where 85% of water was added to mimic the fresh sample. Error bars represent standard deviation for n=3. 12

Fig. S4: Comparisons of chromatographic elution times of interfering compounds and analytes identified with ultra-high performance liquid chromatography and high resolution mass spectrometry. 13

Fig. S5: Stability of anthocyanins monitored in berry smoothie drinks stored in the fridge. Error bars represent standard deviation for n=3. 14

Fig. S6: Degradation of rate of cyanidin 3-O glucoside compared at three storage temperatures after fitting with first order reaction kinetics model. 15

Fig. S7: Stability of flavonoids monitored in berry smoothie drinks stored in the freezer. Error bars represent standard deviation for n=3. 16

Fig. S8: Stability of flavonoids monitored in berry smoothie drinks stored in the fridge. Error bars represent standard deviation for n=3. 17

Fig. S9: Stability of flavonoids monitored in berry smoothie drinks stored at room temperature. Error bars represent standard deviation for n=3. 18

Fig. S10: pH stabilities of berry smoothie drink reported at different storage temperatures. Error bars represent standard deviation for n=3. 19

Fig. S11: Detection of new peak with lambda max 248 after EFA on augmented chromatographic data matrix from room temperature storage. Colum wise augmentation was done on data matrix from baseline samples to 1 month storage. 20

Fig. S12: Chromatographic peaks and spectral profile of compounds observed among samples stored at room temperature for 6 to 9 months. These peaks were not observed or were relatively low in the baseline sample. ..21

S1

The HPLC was transferred to a UHPLC-MS method, of which the settings were modified based on an online waters calculator (Waters, 2019). The UHPLC methods were as follows; The mobile phase consisted of (A) water with 0.1 % formic acid and (B) acetonitrile with 0.1 % formic acid. Based on the online waters calculator, the applied gradient was as follows: 0–1.73 min (5% B); 1.73–3.35 min (5-20 % B); 3.35–4.97 min (20 % B); 4.97–5.94 min (20-25%B); 5.94–6.59 min (25%B); 6.59-7,56 (25-70) 7.56–8.21 min (70%B) and 8.21-14.68 min (70-100% B). A flow rate of 0.4 mL/min and a column temperature of 55 °C were used. The column was a Waters ACQUITY UPLC CSH C18 100 x 2.1 mm, 1.7 µm particle size and an injection volume of 3 µL was set. The masses of compounds were also confirmed using a mass spectrometer (XEVO-G2 QTOF) with electrospray ionization (Waters, MS Technologies, Manchester, UK) employed with the following settings: both positive and negative acquisition mode, mass range (m/z) was 100–1200 Da; capillary voltage, 3 kV (2.5 kV for negative mode); cone voltage, 40 V; source temperature, 120 °C; desolvation temperature, 300 °C; cone gas flow rate, 50 L/h; and desolvation gas flow rate, 600 L/h. MS/MS data was acquired through data dependence acquisition employing collision energy range between 15-65 kV. MS-DIAL (Ver 4.9.221218) was used for MS data deconvolution and treatment. The peaks were also screened for annotation through the help of various databases in MS-DIAL, i.e. Fihn/Vaniya natural product library, RESPECT and Mass Bank.

Table S1:Confirmation of identified analytes in berry smoothie samples using high resolution mass spectrometry (HRMS).

All the mass shown are $[M-H]^-$ form of the compound. with exception of masses marked with * which represent $[M+H]^+$ obtained in positive mode. while ** shows M-2H aglycone form and t_R is retention time. Some compounds shown here were not quantified as they were below limit of quantification in the diode array detector.

t_R in min HPLC (UHPLC t_R)	Detection wavelength (nm)	Analyte Detected	Theoretical mass	Observed mass
8.9 (1.22)	280	Gallic acid	169.0215	169.0144
25.5 (3.61)	280	Catechin	289.0790	289.0707
27.9 (3.66)	325	Chlorogenic acid	353.0951	353.088
30.2 (4.11)	325	Caffeic acid	179.0423	179.0391
36.3 (4.23)	280	Epicatechin	289.0790	289.0718
41.9 (5.14)	325	Ferulic acid	193.0591	193.0501
47.4 (5.88)	325	p-Coumaric acid	163.0473	163.0401
48.4 (5.96)	360	Rutin	609.1534	609.145
49.1(6.25)	360	Quercetin 3-O glucoside	463.0955	463.0896
50.9 (6.38)	360	Kaempferol 3-O glucoside	447.1006	447.093
51.3 (6.90)	325	Resveratrol	227.0786	227.071
51.9 (7.03)	360	Myricetin	317.0376	317.0302

53.8 (7.61)	360	Quercetin	301.0427	301.0334
30.5 (3.05)	520	Delphinidin 3-O glucoside	463.0875	463.0877
35.9 (3.22)	520	Cyanidin 3-O glucoside	449.1079*	449.1077*
39.4 (3.63)	520	Petunidin 3-O glucoside	479.1189*	479.1187*
40.7 (3.75)	520	Pelargonidin 3-O glucoside	433.1134*	433.1133*
43.1 (3.88)	520	Peonidin 3-O glucoside	299.0712**	299.0553
44.4 (4.01)	520	Malvidin 3-O glucoside	492.1346	492.123

Table S2: Molar absorptivity of different compounds measured at different wavelengths.

Maleic acid and tryptophan represent the organic acid and amino acids detected as interferences in this study, while other compounds represent different classes of polyphenols

	Molar absorptivity (L. mol^{-1}. cm^{-1})			
**Compound**	**280 nm**	**325 nm**	**360 nm**	**520 nm**
Maleic acid (organic acid)	38303	1149	743	NA
Tryptophan (amino acid)	633113	653	408	NA
Gallic acid (phenolic acid)	1922356	34024	8166	136
Chlorogenic acid (phenolic acid)	1027499	1948705	389741	2267
Quercetin (flavonoid)	2871280	3717552	6800400	2478
Cyanidin 3O glucoside (anthocyanin)	3145660	179752	161776	1572830

Table S3: Interfering compounds of identified analytes in berry smoothie samples using high resolution mass spectrometry (HRMS).

All the mass shown are $[M-H]^-$ form of the compound, with exception of masses marked with * which represent $[M+H]^+$ obtained in positive mode.

t_R (UHPLC)	Suggested compound	Observed m/z (M-H)	Theoretical mass (M-H)
Organic acids			
0.66	2-Oxobutyric acid	101.02415	101.03169
0.72	Ascorbic acid	175.02455	175.03209
0.73	Quinic acid	191.05676	191.06339
0.82	L-()-Tartrate	149.00899	149.01644
0.82	Maleic acid	115.00351	115.01095
0.82	Malic acid	133.01418	133.02152
0.94	Citric acid	191.02003	191.02700
Amino acids			
0.503	L-Arginine	175.11954*	175.11167*
0.503	L-Proline	116.07131*	116.06332*
3.61	Tryptophan	203.06799	203.08987
Other heterocyclic compounds			
2.099	Hydroquinone	109.02753	109.036779
2.134	Hematein	299.07623	299.06339
4.029	Niacinamide	121.02956	121.048012
4.046	3-Cyanopyridine	105.03426*	105.037448*
4.234	Columbianetin	245.08162	245.08921

4.925	2-hydroxy-4-(hydroxymethyl)-6-(1-hydroxy-3-methylbut-2-enyl)-3-[(E)-prop-1-enyl]-7-oxabicyclo[4.1.0]hept-3-en-5-one	279.12497	279.131073
5.728	Eudesmin	385.14832	386.172938
5.889	Coumarin	147.04379*	147.03679*
Terpenes			
7.691	2-[[3,4-dihydroxy-4-(hydroxymethyl)oxolan-2-yl]oxymethyl]-6-[(6,6-dimethyl-2-bicyclo[3.1.1]hept-2-enyl)methoxy]oxane-3,4,5-triol	445.20218	445.21519
6.061	7-dimethyl-7-(4-methylpent-3-enyl) bicyclo [2.2.1]heptan-3-ol	220.95511	221.19836
6.727	Colforsin	409.2085	409.23045

Table S4: Kinetic parameters for degradation of anthocyanins derived from first order fitting model.

RT stand for room temperature.

Compound	Storage condition	Rate constant *k* (month-1)	Half-life, $t_{1/2}$ (month)	R^2	RMSE
**Cyanidin 3-0 glucoside**	RT	0.1	7	0.9029	1.2
	Fridge (4 °C)	0.02	29	0.8024	1.4
	Freezer (-20 °C)	0.02	28	0.9325	1.8
**Delphinidin 3-O glucoside**	RT	0.06	12	0.9691	1.8
**Petunidin 3-0 glucoside**	RT	0.07	10	0.9667	0.5
**Pelargonidin 3-0 glucoside**	RT	0.06	12	0.7122	0.8
**Peonidin 3-0 glucoside**	RT	0.08	8	0.9114	0.8
**Malvidin 3-0 glucoside**	RT	0.09	7	0.9640	0.6

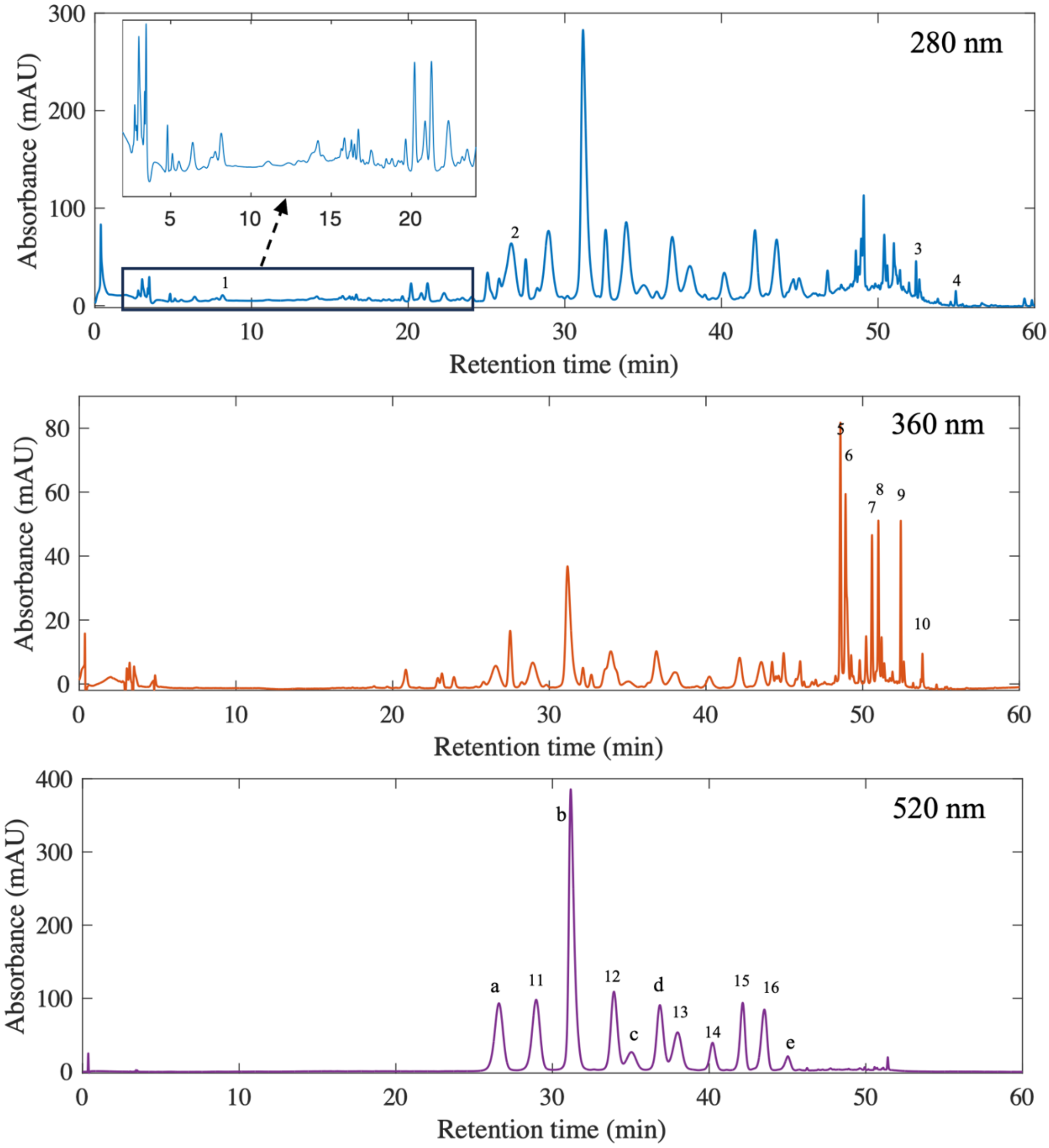

Fig. S1: Chromatogram of berry smoothie sample at three different wavelengths.

280 nm (1. Gallic acid. 2. Chlorogenic acid. 3. Resveratrol (Stilbene). 4. Trans-cinnamic acid); 360 nm (5. Rutin. 6. Quercetin 3-O glucoside. 7. Kaempferol 3-O glucoside. 8. Myricetin. 9. Quercetin. 10. Kaempferol); 520 nm (11. Delphinidin 3-O glucoside. 12. Cyanidin 3-O glucoside. 13. Petunidin 3-O glucoside. 14. Pelargonidin 3-O glucoside. 15. Peonidin 3-O glucoside. 16. Malvidin 3-O glucoside). Peak labelled a. b. c. d and e. are unknown

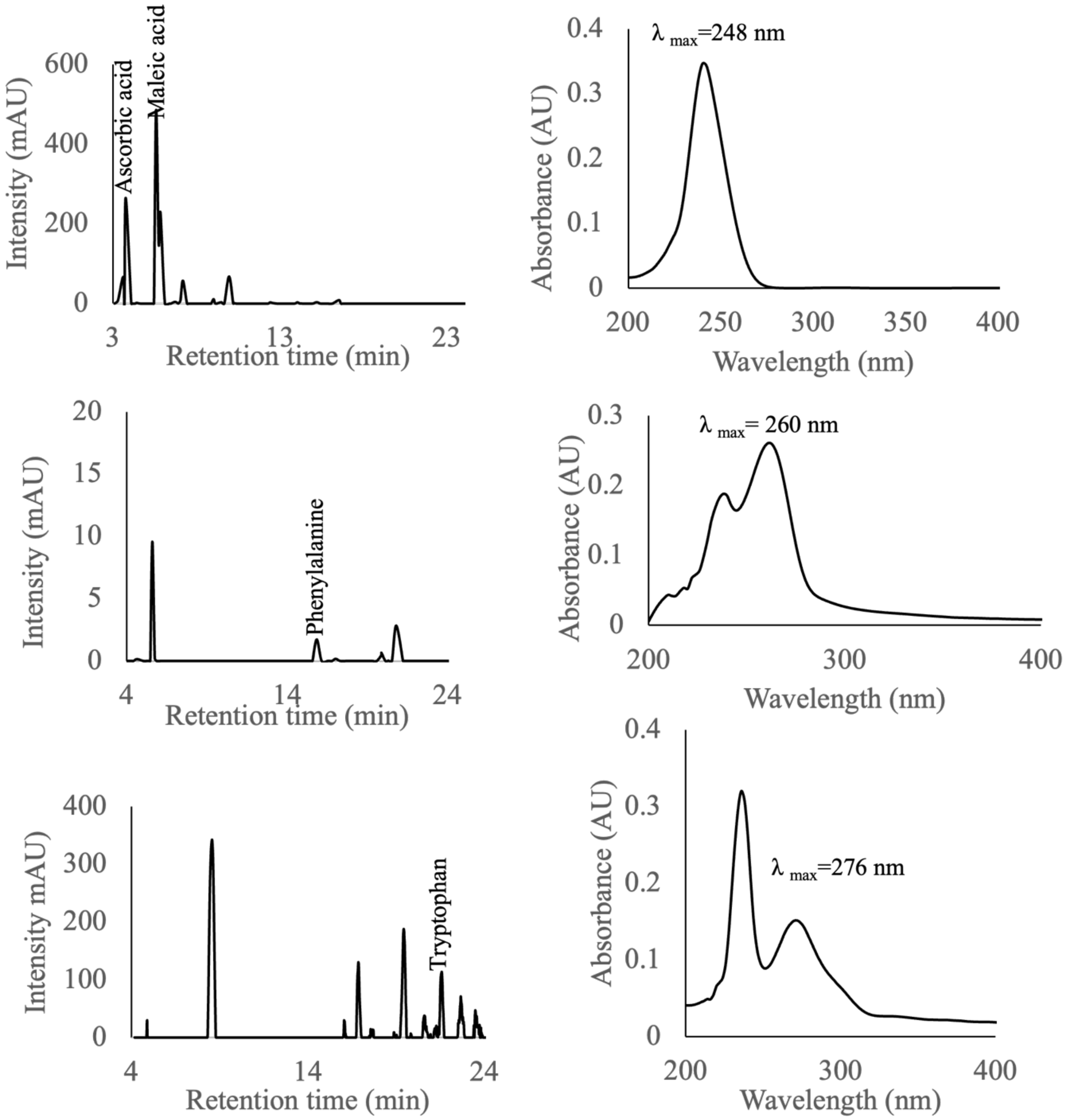

Fig. S2: Interfering organic acids and amino acids identified with standards after HPLC-DAD and MCR -ALS data processing

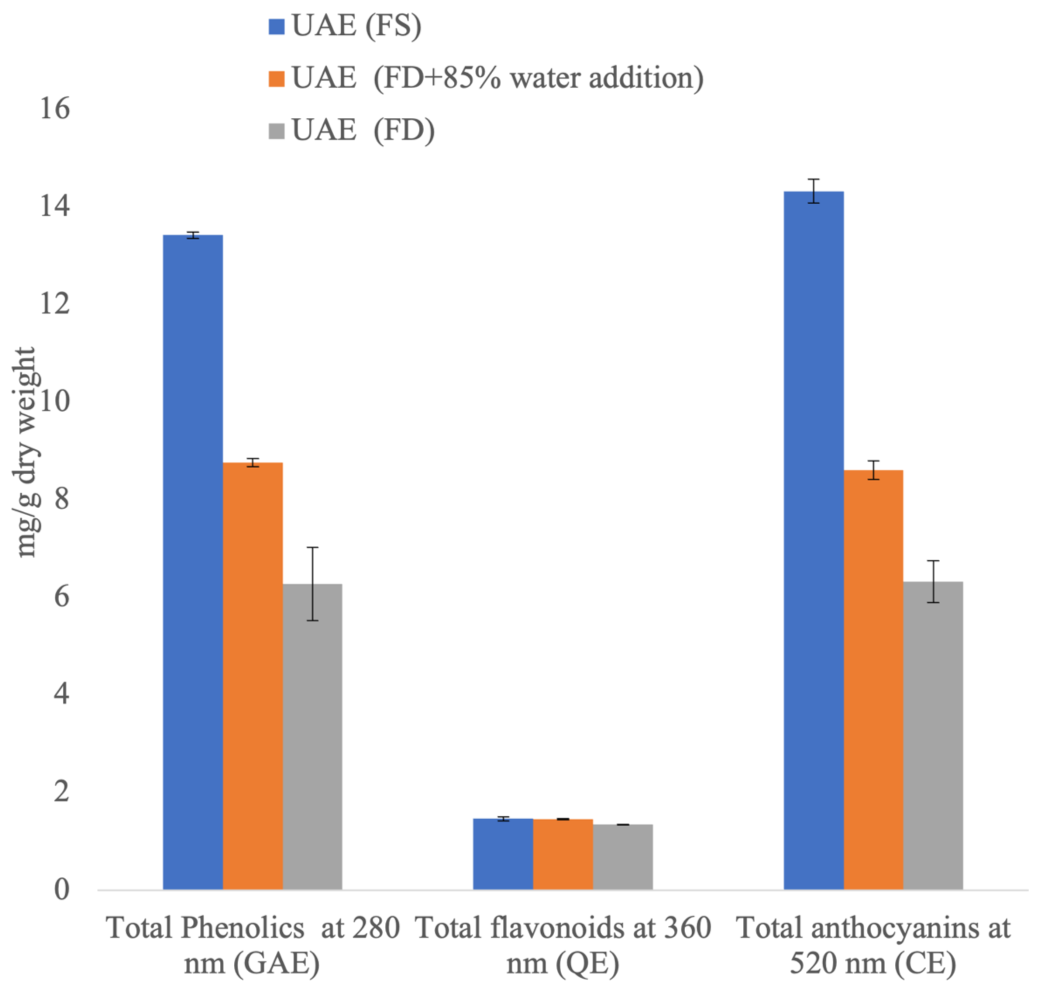

Fig. S3: Total phenolic, total flavonoids and total anthocyanin content extracted using UAE with acidified MEOH, both original fresh sample and freeze-dried sample where 85% of water was added to mimic the fresh sample. Error bars represent standard deviation for n=3.

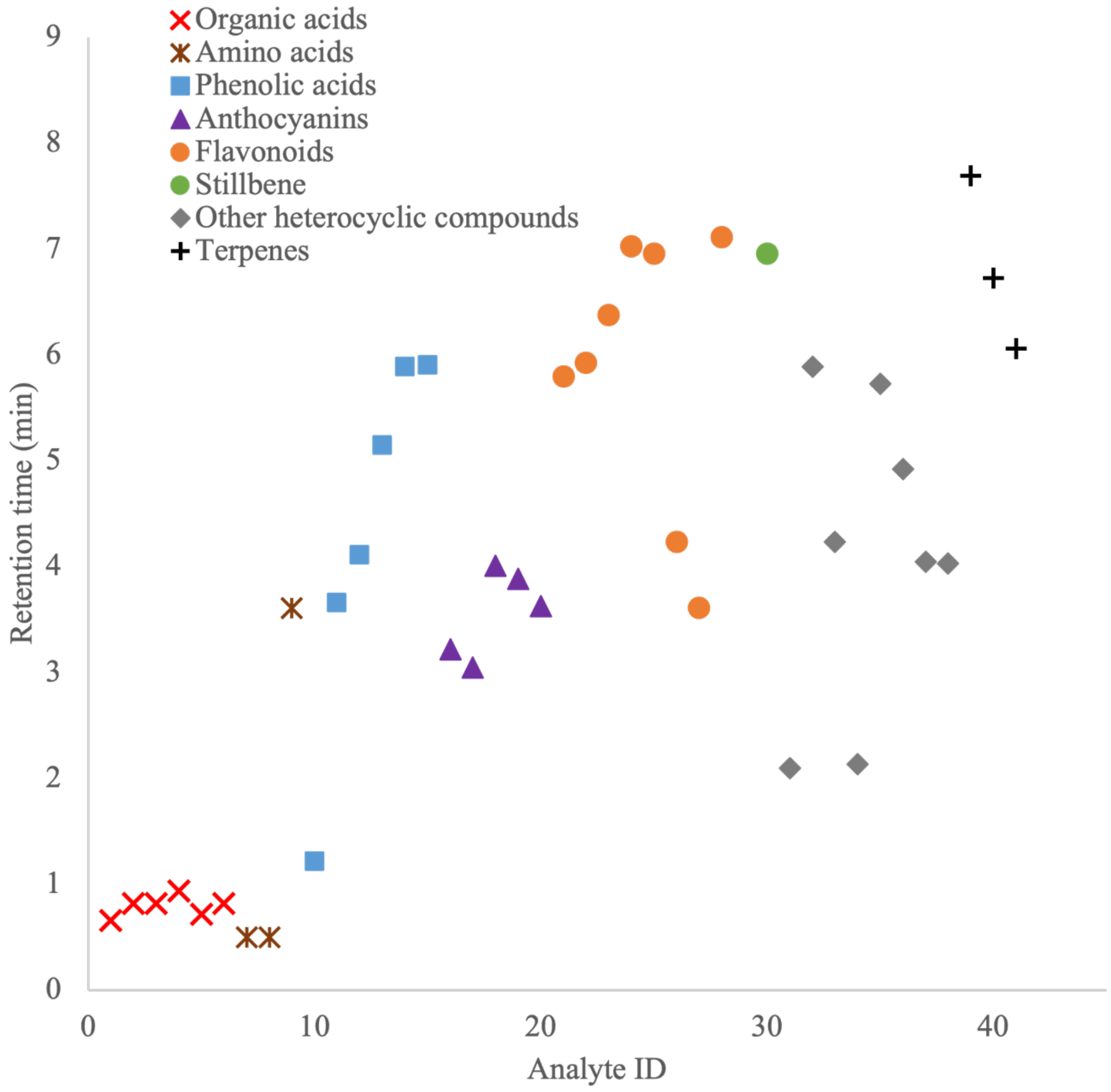

Fig. S4: Comparisons of chromatographic elution times of interfering compounds and analytes identified with ultra-high performance liquid chromatography and high resolution mass spectrometry.

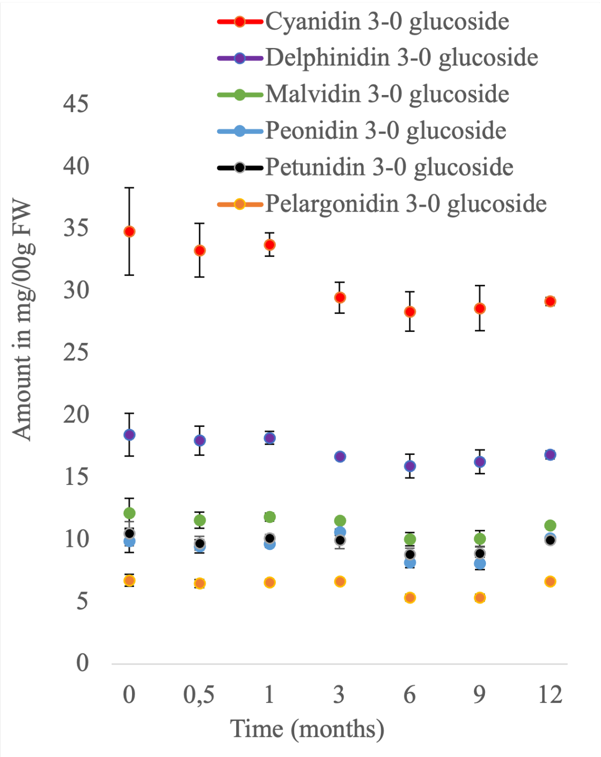

Fig. S5: Stability of anthocyanins monitored in berry smoothie drinks stored in the fridge. Error bars represent standard deviation for n=3.

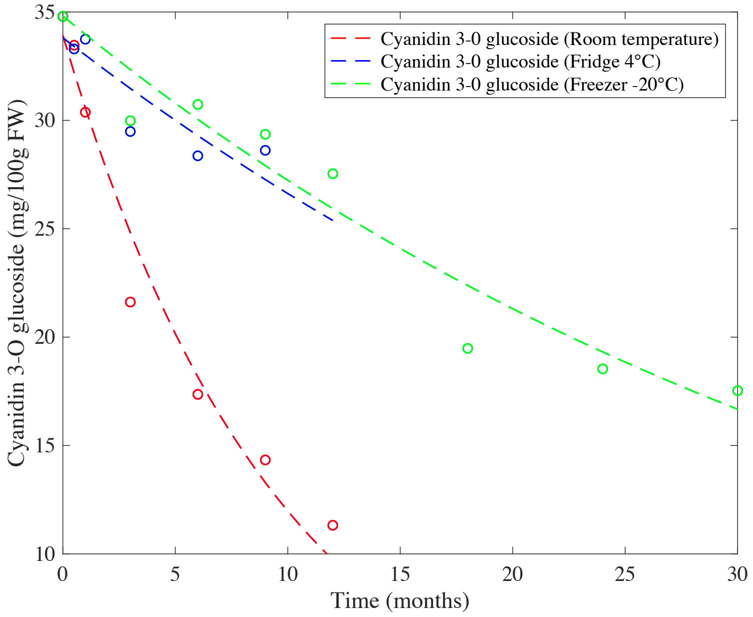

Fig. S6: Degradation of rate of cyanidin 3-O glucoside compared at three storage temperatures after fitting with first order reaction kinetics model.

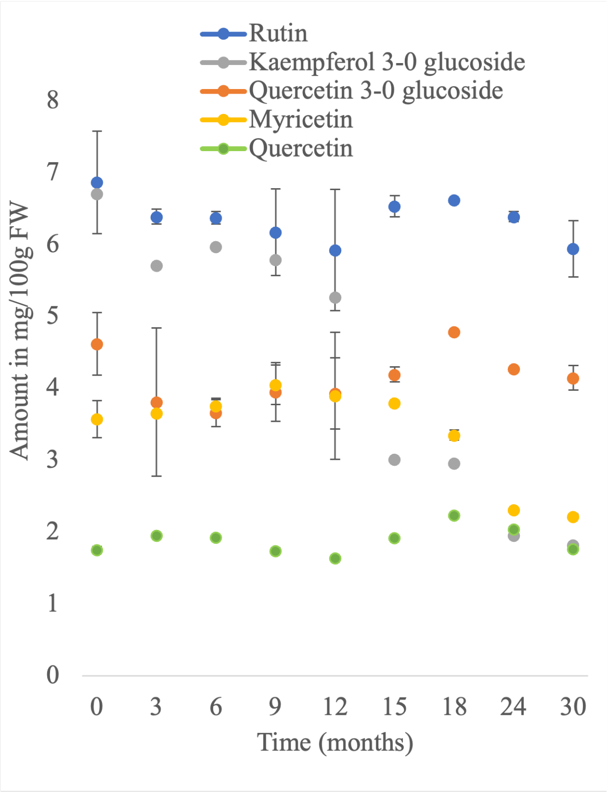

Fig. S7: Stability of flavonoids monitored in berry smoothie drinks stored in the freezer. Error bars represent standard deviation for n=3.

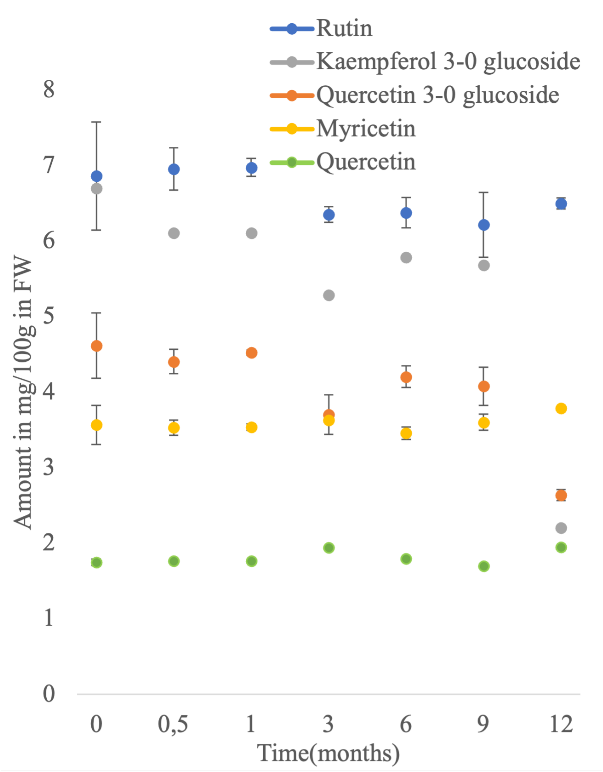

Fig. S8: Stability of flavonoids monitored in berry smoothie drinks stored in the fridge. Error bars represent standard deviation for n=3.

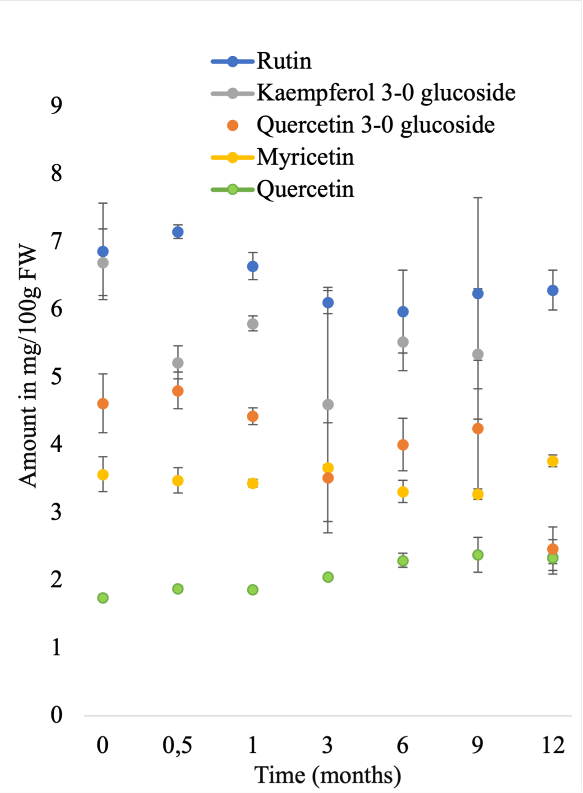

Fig. S9: Stability of flavonoids monitored in berry smoothie drinks stored at room temperature. Error bars represent standard deviation for n=3.

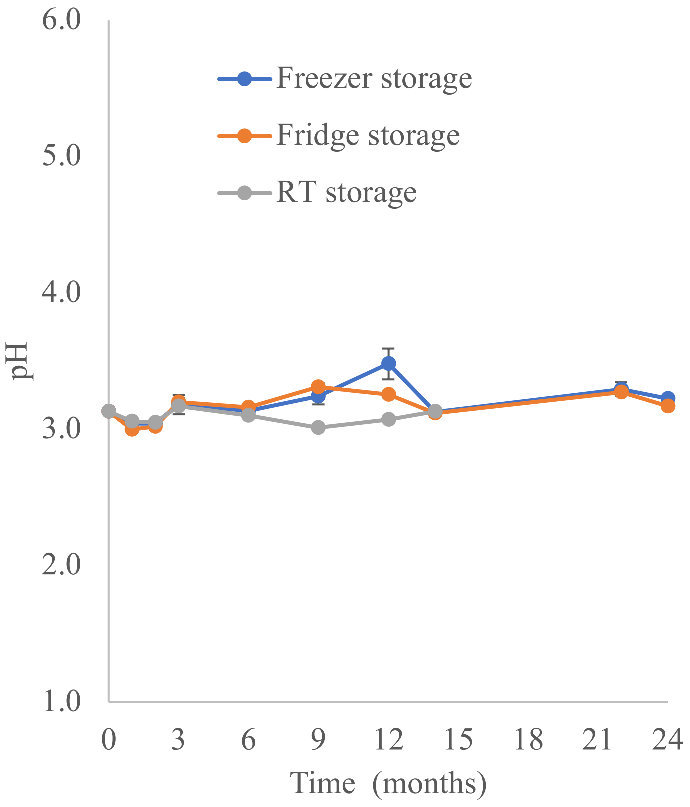

Fig. S10: pH stabilities of berry smoothie drink reported at different storage temperatures. Error bars represent standard deviation for n=3.

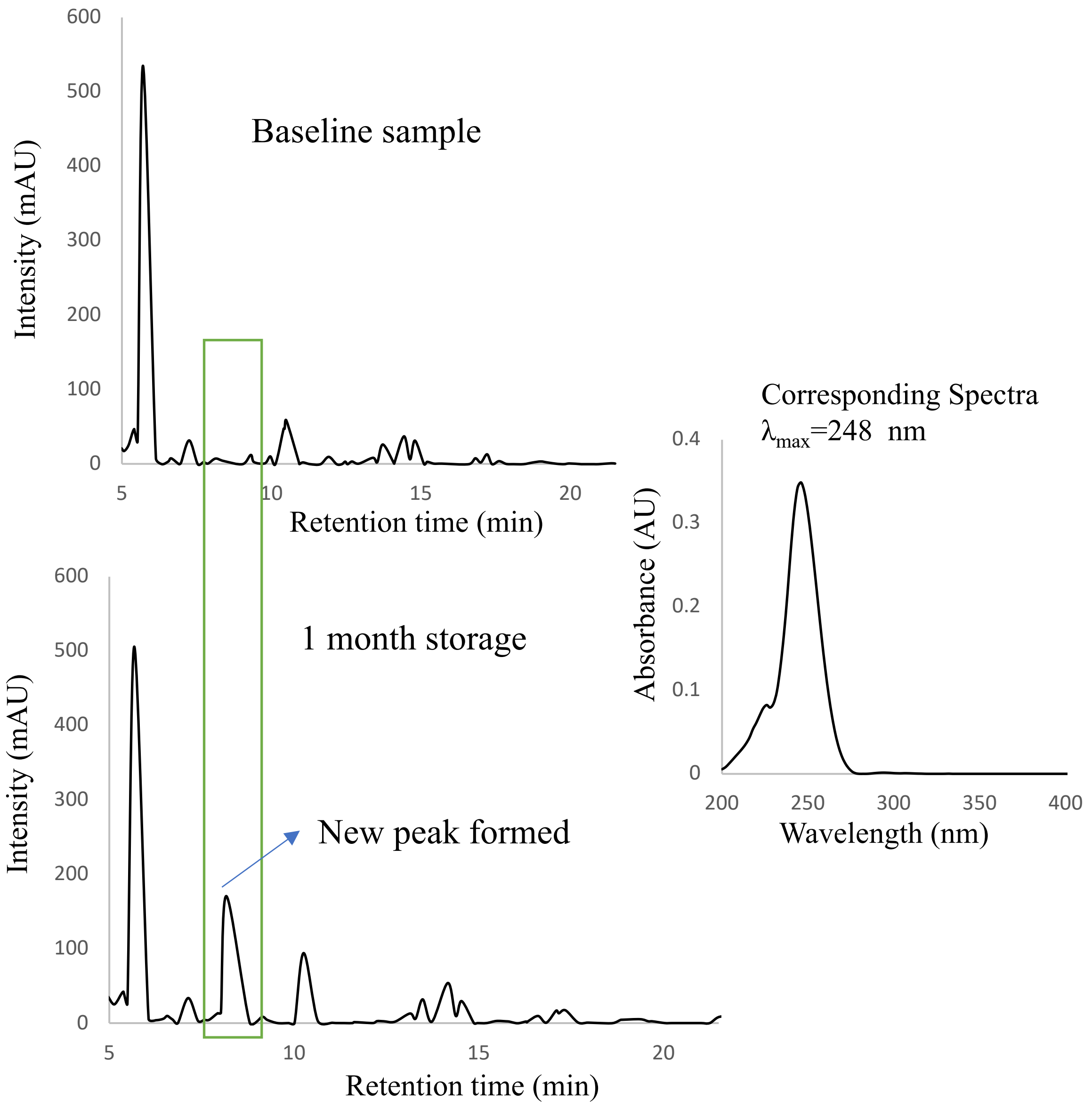

Fig. S11: Detection of new peak with lambda max 248 after EFA on augmented chromatographic data matrix from room temperature storage. Colum wise augmentation was done on data matrix from baseline samples to 1 month storage.

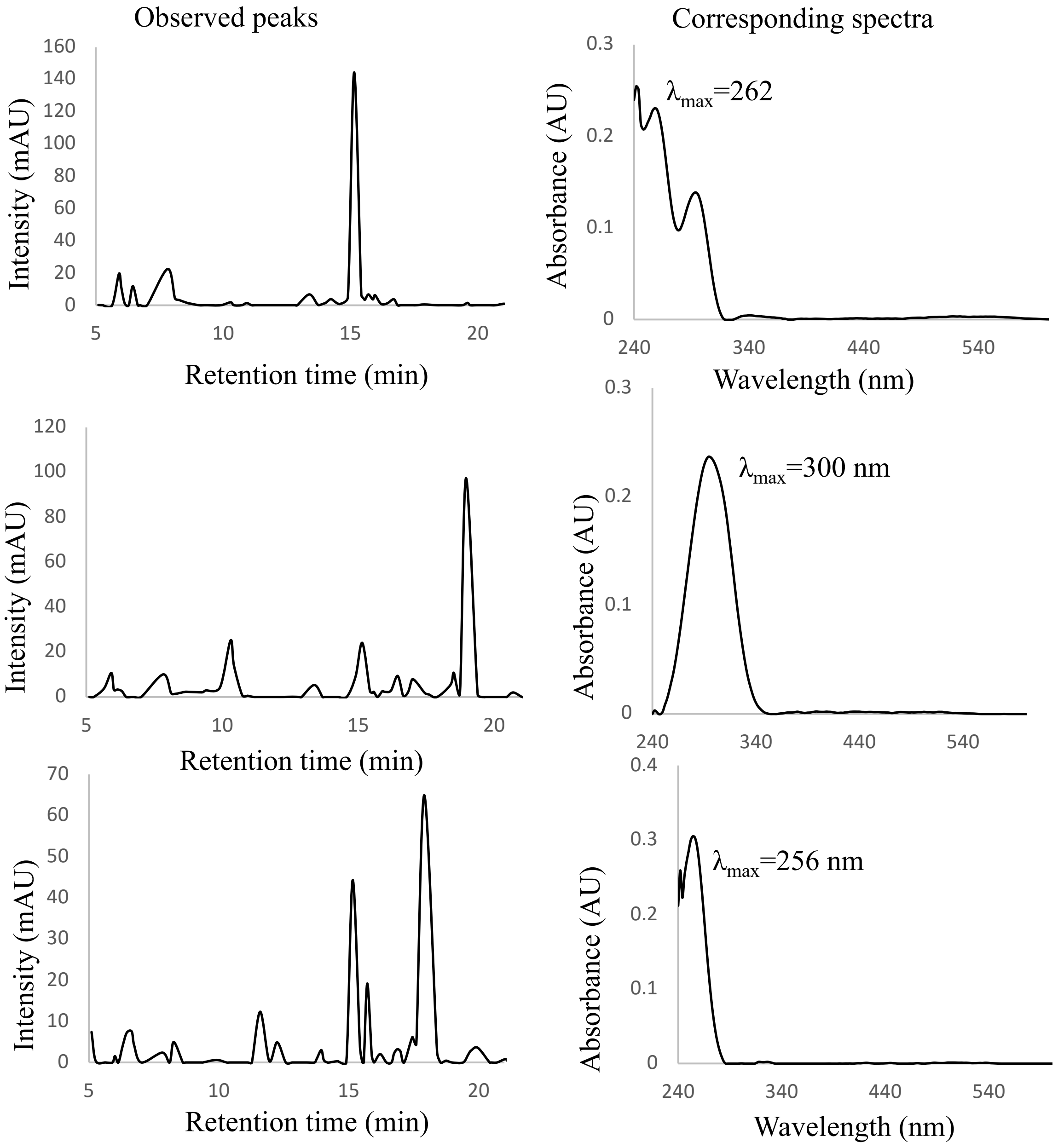

Fig. S12: Chromatographic peaks and spectral profile of compounds observed among samples stored at room temperature for 6 to 9 months. These peaks were not observed or were relatively low in the baseline sample.
